# Functional and genomic characterization of LCN2-deficient PC-3 cells reveals insights into prostate cancer progression

**DOI:** 10.3389/fmolb.2026.1730948

**Published:** 2026-04-20

**Authors:** Kiara Gäberlein, Diandra T. Keller, Sarah K. Schröder-Lange, Phillipp Torkler, Dominik G. Grimm, Claus Steinlein, Thomas Haaf, Indrajit Nanda, Ralf Weiskirchen

**Affiliations:** 1 Institute of Human Genetics, Julius Maximilians University of Würzburg, Würzburg, Germany; 2 Institute of Molecular Pathobiochemistry, Experimental Gene Therapy and Clinical Chemistry (IFMPEGKC), RWTH University Hospital Aachen, Aachen, Germany; 3 Faculty of Computer Science, Deggendorf Institute of Technology, Deggendorf, Germany; 4 TUM Campus Straubing for Biotechnology and Sustainability, Technical University of Munich & Weihenstephan-Triesdorf University of Applied Sciences, Straubing, Germany; 5 Institute of Clinical Genetics and Genomic Medicine, University Hospital Würzburg, Würzburg, Germany

**Keywords:** cell culture model, chromosome analysis, CRISPR/Cas9, gene disruption, LCN2, lipocalins, PC-3, prostate cancer

## Abstract

**Introduction:**

Prostate Cancer-3 (PC-3) cells, commonly used as a model for aggressive, androgen-independent prostate cancer, display numerous genetic alterations that contribute to advanced disease, including the loss of tumor suppressors and dysregulated inflammatory signaling. Recent evidence has highlighted the pleiotropic roles of lipocalin 2 (LCN2) in promoting tumor cell proliferation, adhesion, and stress resistance. This study aimed to investigate the functional and molecular effects of *LCN2* depletion in PC-3 cells.

**Methods:**

We conducted a genetic analysis of both the parental PC-3 cell line and a newly created *LCN2*-deficient PC-3 clone #1 (PC-3 LCN2-KO#1), developed using CRISPR/Cas9 technology. Short tandem repeat (STR) analyses confirmed the authenticity and lineage of each cell line, while next-generation sequencing coupled with RT-qPCR validation was used to identify differentially expressed genes and any potential genomic changes resulting from the CRISPR/Cas9 editing process.

**Results and Discussion:**

Our analysis aligned with our previous findings showing that *LCN2* is involved in inflammation, endoplasmic reticulum stress responses, and cytoskeletal organization. Previously we have shown that *LCN2*-deficient cells exhibited decreased invasiveness, disrupted F-actin dynamics, and increased sensitivity to stress-inducing conditions. Consistent with these observations, spectral karyotyping (SKY) and analysis of spontaneously occurring micronuclei revealed an elevated level of chromosomal aberrations in the *LCN2*-deficient cell line. These results emphasize the significance of *LCN2* in driving prostate cancer aggressiveness and provide a foundation for exploring targeted interventions that disrupt *LCN2*-mediated pathways in advanced disease.

## 1 Introduction

Prostate Cancer-3 (PC-3) cells were established in 1979 by Kaighn and colleagues from a bone metastasis in a 62-year-old patient with advanced grade IV prostate adenocarcinoma (Kaighn et al., 1979). These cells were developed to study the characteristics of aggressive, androgen-independent prostate cancer. They do not depend on androgens for growth, making them a valuable model for late-stage disease and offering insights into hormone-refractory conditions (Kaighn et al., 1979; Rudzinski et al., 2020).

Since they were isolated from a metastatic lesion, PC-3 cells naturally have altered regulatory mechanisms that promote aggressive traits, such as rapid growth and migratory behavior. Specifically, these cells produce several cytokines and growth factors that can potentially stimulate tumor innervation (Voss et al., 2010). Consequently, they have become essential in prostate cancer research, frequently used in drug screening, co-culture experiments, and *in vivo* models to explore therapeutic strategies for managing bone metastases. The cells are also utilized in xenograft models, with the ability to induce lymph node metastases (Wu et al., 2013).

Notably, PC-3 cells lack androgen receptors, which explains their unresponsiveness to androgen-deprivation therapies. However, other studies have shown the existence of a subline of PC-3 cells that produce measurable androgen receptor mRNA and traces of AR protein (Buchanan et al., 2004). They also contain genetic alterations like the loss of the tumor suppressor *PTEN* and deletions, as well as abnormalities in *TP53* (Fraser et al., 2012; Chappell et al., 2012; Seim et al., 2017). Genome-wide analyses indicate that inflammation- and proliferation-related pathways are upregulated in these cells, further emphasizing their aggressive phenotype compared to other human prostate cancer cell lines (Dozmorov et al., 2009). Altogether, these features make PC-3 cells an indispensable tool for understanding the molecular basis of advanced prostate cancer and for guiding the development of innovative therapeutic strategies. Although they have been extensively studied, ongoing research continues to refine our understanding of their biology, highlighting the importance of verifying specific experimental details with the original literature.

Another recent significant application of the PC-3 cell line involves generating *LCN2*-deficient variants to study how this secreted protein influences prostate cancer behavior (Schröder et al., 2022). LCN2, also known as neutrophil gelatinase-associated lipocalin (NGAL), is a 25-kDa transporter protein belonging to a large family of small extracellular proteins involved in various cellular processes, including innate immunity, cellular adhesion, inflammatory signaling, and tumor progression (Asimakopoulou et al., 2016; Schröder et al., 2023a; Chandrasekaran et al., 2024). In a previous study, CRISPR/Cas9-mediated knockout and siRNA-based suppression of *LCN2* in PC-3 cells were used to understand how *LCN2* deficiency impacts metastatic properties (Schröder et al., 2022). Remarkably, *LCN2*-deficient PC-3 cells showed reduced proliferation and cell adhesion, disrupted F-actin organization, decreased expression of pro-inflammatory cytokines, and increased sensitivity to endoplasmic reticulum stress. Reduced IL-1β expression, along with decreased invasiveness and compromised stress response, further underscored the significance of *LCN2* in advanced prostate cancer. These findings collectively demonstrate the usefulness of PC-3-based *LCN2*-knockout models in dissecting the molecular pathways involved in prostate cancer progression and validating *LCN2* as a promising therapeutic target in aggressive disease stages (Schröder et al., 2022). Furthermore, disrupting *LCN2* in these cells led to decreased activation of the JAK/STAT pathway and reduced expression of interferon-stimulated genes (Barer et al., 2023). All these findings suggest that comparing parental PC-3 cells with *LCN2*-deficient subclones derived from them is a valuable tool for investigating aspects of *LCN2* function in prostate cancer.

In this paper, we will genetically characterize one of our newly generated PC-3 clones, designated as clone #1, and compare its mRNA and protein expression profile to that of the parental PC-3 cell line. Additionally, we conducted next-generation sequencing (NGS) on clone #1 and the parental PC-3 cell line to systematically evaluate gene expression changes in the absence of *LCN2*. This approach enabled us to identify molecular pathways that are differentially regulated when *LCN2* is depleted, providing more insights into the role of lipocalins in prostate cancer progression. Furthermore, SKY analysis was used to compare the genome wide structural organization of clone #1 with that of the parental PC-3 cell line.

## 2 Materials and methods

### 2.1 Literature search

A comprehensive review of relevant literature was conducted to identify studies using the PC-3 prostate cancer cell line, focusing on its genetic alterations, protein expression, and functional assays. Databases searched included PubMed, Web of Science, and Scopus. The search strategy involved combining the terms “PC-3,” “prostate cancer,” and “cell line,” along with “genetic,” “expression,” or “knockout.” Priority was given to articles published in English, and reference lists of key studies were examined to locate additional relevant publications. The selected papers informed both the experimental design and the interpretation of our results. Additionally, we explored the FDI Lab: SciCrunch Infrastructure database for references to PC-3 (SciCrunch).

### 2.2 Cell culture

PC-3 cells (CRL-1435; RRID:CVCL_0035) were obtained from the German branch (LGC Standards GmbH, Wesel, Germany) of the American Type Culture Collection (ATCC). Both, the parental PC-3 cells and the previously established clone #1 of PC-3 lacking *LCN2* (referred to as PC-3 LCN2-KO#1) (Schröder et al., 2022) were cultured in Dulbecco’s Modified Eagle Medium (DMEM, #D6171, Sigma-Aldrich, Merck, Taufkirchen, Germany) supplemented with 10% (v/v) fetal bovine serum (FBS, #F7524, Sigma-Aldrich), 2 mM L-glutamine (#G7513, Sigma-Aldrich), and 1× penicillin/streptomycin (DE17-602E, Lonza, Cologne, Germany). The cultures were maintained at 37 °C in a humidified atmosphere with 5% CO_2_ and split when they reached 70%–80% confluency. For subculturing, the cells were rinsed with phosphate-buffered saline (PBS) and detached using Accutase solution (#A6964-100ML, Sigma-Aldrich).

### 2.3 Light microscopy, FACS analysis, and phalloidin stain

Live-cell morphology was observed using a Leica DM IL LED microscope equipped with a Leica EC3 camera and the Leica Application Suite (LAS) software (version 3.4.0), all from Leica (Wetzlar, Germany), with phase-contrast, bright field, or fluorescent illumination. Images were captured using an attached digital camera with bright field or fluorescent illumination. Bright-field images were used to assess cell density, confluency, and any morphological changes indicating contamination or differentiation. For FACS analysis, both cell lines were grown in 10 cm plates for 2 days, until they reached approximately 80% confluency. Cells were then detached using Accutase® solution, collected by adding medium, and centrifuged at 300 rpm for 8 min at 4 °C. After discarding the supernatant, the resulting pellets were gently suspended in 1 mL of FACS buffer (10 mM HEPES, pH 7.3, 0.06% bovine serum albumin, and 0.3 mM EDTA) prepared in Hank’s balanced salt solution without phenol red, calcium and magnesium (#88284, Thermo Fisher Scientific). The samples were placed on ice, filtered through a 40 µm nylon mesh into dedicated FACS tubes, and briefly re-suspended before analysis. Flow cytometry was performed on a BD FACSAria II SORP, with BD FACSDiva™ 6.0 software used to process 10,000 events per sample. A forward scatter (FSC) threshold of 5,000 was set to exclude debris, and doublet discrimination was applied to the side scatter (SSC) and FSC channels. The proportion of GFP-positive cells was then recorded. Phalloidin staining of PC-3 cells and their derivatives was performed using a protocol that we have described before (Schröder et al., 2023b) and images were taken with a Nikon Eclipse E80i fluorescence microscope (Nikon Imaging Japan Inc., Shinagawa-ku, Tokyo, Japan).

### 2.4 Short tandem repeat profiling

To verify the authenticity of both parental PC-3 and clone #1 cells, short tandem repeat (STR) profiling was conducted at IDEXX Laboratories in Kornwestheim, Germany. The CellCheck™ Human 16 STR Profile and Interspecies Contamination Test were used for this purpose. The resulting STR profiles were then compared using the online Cellosaurus STR similarity search tool (CLASTR, version 1.4.4) (Cellosaurus CLASTR, 2025) with the following search parameters: Scoring: Tanabe; Modes: Non-empty markers; Filters: Score Filter: 60%, Min Markers: 8; Max Results: 200. In the Tanabe scoring system, also known as Sørensen-Dice coefficient, 15–17 polymorphic STR markers (typically 2-6 base pairs) are used to generate a profile that verifies cell line identity, ancestry, and sex (Amelogenin) (Tanabe et al., 1999).

### 2.5 Next-generation mRNA sequencing and data analysis

Total RNA from both parental PC-3 cells and clone #1 cells, isolated from cells grown to 70% confluence, underwent next-generation sequencing (NGS) to identify differentially expressed genes influenced by *LCN2* deficiency. The RNA samples were initially assessed for concentration, purity, and integrity using UV spectrophotometry and an Agilent 4200 TapeStation (Agilent Technologies Inc., Waldbronn, Germany). After removal of ribosomal RNA, the remaining mRNA fraction was converted into a sequencing library using the NEBNext® Multiplex Oligos for Illumina® Index Primers Set 1 (#E7335S, New England Biolabs, Frankfurt am Main, Germany). These libraries were then sequenced on an Illumina instrument using 300-cycle MiSeq Reagent kit V2 cartridges (Illumina Inc., San Diego, CA, USA). All cDNA library preparations and sequencing procedures were conducted at the IZKF Genomic Facility, University Hospital Aachen. The resulting sequencing runs were demultiplexed and converted to FASTQ format using Illumina bcl2fastq conversion software (version 2.20), and these FASTQ files were utilized for subsequent downstream analyses. Raw sequencing reads were assessed for quality using FastQC (v0.11.8) (Babraham). Following the evaluation, adapters and bases with Phred scores below 20 were eliminated. Any reads shorter than 35 bp were discarded using cutadapt (v1.9.1) (Martin, 2011), and reads were aligned to the reference genome using STAR (v2.7.11a) (Dobin et al., 2013). Gene abundances were then estimated using featureCounts (v2.0.6) and the pseudoalignment-based methods kallisto (v0.48.0) (Bray et al., 2016) and salmon (v.1.10.2) (Patro et al., 2017), followed by median-of-ratios normalization (Love and Huber, 2014). For annotation purposes, the Human GRCh38.p14 reference transcriptome (Ensembl) was employed. To generate a candidate list of genes with potentially changed expression upon *LCN2* depletion, which was later verified by RT-qPCR, we filtered out lowly expressed genes and only retained genes with log2 fold-changes ≥2 as estimated by featureCounts, kallisto, and salmon.

### 2.6 RNA extraction and real-time quantitative PCR

For targeted validation of selected differentially expressed genes, total RNA was extracted using the PureLink RNA Mini kit (#12183018A, Thermo Fisher Scientific Inc., Life Technologies GmbH, Darmstadt, Germany) following the manufacturer’s instructions. RNA concentration and purity were assessed using a NanoDrop 2000 spectrophotometer (Thermo Fisher Scientific). First-strand cDNA was synthesized from 1 µg of total RNA using Superscript II Reverse Transcriptase (#18064–014, Thermo Fisher Scientific) with random hexamer primers (#C118A, Thermo Fisher Scientific). The resulting cDNA was diluted in RNase-free water and stored at −20 °C. For quantitative real-time PCR (RT-qPCR), 5 µL of diluted cDNA was combined with sequence-specific primers in a 25 µL reaction containing SYBR-Green™ qPCR SuperMix (#56465, Thermo Fisher Scientific). The thermal cycling protocol included an initial 10-min denaturation step at 95 °C, followed by 40 cycles of 15 s at 90 °C and 1 min at 60 °C. RT-qPCR measurements were performed in technical duplicates for each biological sample. The experiment was independently repeated three times, resulting in biological triplicates (n = 3). Expression levels were normalized to glyceraldehyde 3-phosphate dehydrogenase (*GAPDH*). Relative mRNA abundance was calculated using the 2^−ΔΔCT^ method (Schmittgen and Livak, 2008). All primers used in the study are listed in Supplementary Table S1.

### 2.7 Western blot analysis

Protein expression levels were determined using Western blot analysis. Cells were lysed in radioimmunoprecipitation assay (RIPA) buffer, which consisted of 20 mM Tris-HCl (pH 7.2), 150 mM NaCl, 2% (w/v) NP-40, 0.1% (w/v) SDS, 0.5% (w/v) sodium deoxycholate, and the cOmplete™ protease inhibitor cocktail (#CO-RO, Merck, Darmstadt, Germany). Protein concentrations were measured using the DC Protein Assay (Bio-Rad Laboratories GmbH, Feldkirchen, Germany). Equal amounts of protein (40 µg) were separated by 10% SDS-PAGE under reducing conditions and transferred to 0.45 µm nitrocellulose membranes (GE Healthcare, Buckinghamshire, UK). Ponceau S stain was used to confirm successful protein transfer. The membranes were blocked with 5% (w/v) non-fat dry milk in Tris-buffered saline (50 mM Tris, pH 7.6, 150 mM NaCl) containing 0.1% Tween 20. Subsequently, membranes were incubated overnight at 4 °C with primary antibodies targeting specific proteins, followed by horseradish peroxidase-conjugated secondary antibodies at room temperature for 1 h. β-Actin was used as a loading control. Signals were detected using the Supersignal™ West Dura Extended Duration Substrate (#34076, ThermoFisher Scientific) and captured with an iBright™ 1500 Imaging System (Invitrogen, Thermo Fisher Scientific). The antibodies utilized in our study are listed in Supplementary Table S2.

### 2.8 Karyotype analysis and spectral karyotype analysis

Standard karyotype and SKY analyses were conducted on metaphase spreads from parental PC-3 and clone #1 cells to evaluate chromosomal composition. Both cell lines were cultured in T25 flasks at 37 °C until reaching semi-confluence. Subsequently, the cells were treated with colcemid, detached using mild trypsin-EDTA, and collected by centrifugation. After a 30-min hypotonic treatment with 0.56% KCl at 37 °C, the cells were fixed in methanol-acetic acid (3:1) solution. Air-dried chromosome spreads were prepared, and chromosome number and morphology were assessed using conventional Giemsa staining.

Chromosome rearrangements in both cell lines were examined using a commercial Human SKY probe from Applied Spectral Imaging in Carlsbad, CA. Slides aged 2–3 days were used for hybridization. Denaturation, *in situ* hybridization, and chromosomal counterstaining were carried out according to the manufacturer’s protocol. Images were captured using a Zeiss microscope AxioImager A1 (Jena, Germany). Spectral classification and pseudo-coloring of chromosomes were performed using HiSky 6.0 software (Applied Spectral Imaging, Carlsbad, CA, USA). At least 20 metaphases were analyzed by SKY for each cell line.

To validate the chromosomal rearrangements detected by SKY analysis, fluorescence *in situ* hybridization (FISH) was performed on metaphase spreads from both cell lines using commercially available chromosome-specific paints (Kreatech, Leica Biosystems, Amsterdam, Netherlands), following established standard protocols.

### 2.9 Data analysis and statistical analysis

Statistical analysis was performed with GraphPad Prism v.8.0 (GraphPad Software, Inc., La Jolla, CA). All data in this study are shown as mean ± standard deviation (SD). Statistical significance between groups was assumed in ANOVA test when probability values were below 0.05 (p < 0.05). The normality of data distribution was assessed using the Shapiro-Wilk test. For data that was normally distributed, statistical significance was evaluated using ordinary one-way ANOVA. For data that did not meet the assumptions of normality, the non-parametric Kruskal–Wallis test was applied. Significant differences are indicated by asterisks: *p < 0.05, **p < 0.01, ***p < 0.001, ****p < 0.0001.

## 3 Results

### 3.1 Usage of PC-3 cells in biomedical research

PC-3 cells are one of the most commonly used cell lines in prostate cancer research due to their androgen-independent status and aggressive metastatic characteristics. They have been cited in the Resource Identification Initiative (RRID) database over 1,000 times, indicating their widespread adoption in biomedical science. A PubMed search using the search terms “PC-3 or PC3” for the time period 1979 to 2025 yielded 17,627 entries (search conducted on 21 October 2025). These entries include studies from various disciplines, with the majority focusing on tumor progression, screening new drugs, and investigating the mechanistic pathways involved in advanced prostate cancer. Similarly, SciCrunch, which provides a curated database of reagents, tools, and materials, has reported a consistent increase in the use of PC-3 in recent years (Supplementary Figure S1).

### 3.2 Short tandem repeat profiling of parental PC-3 and PC-3 LCN2-KO clone #1

**FIGURE 1:**
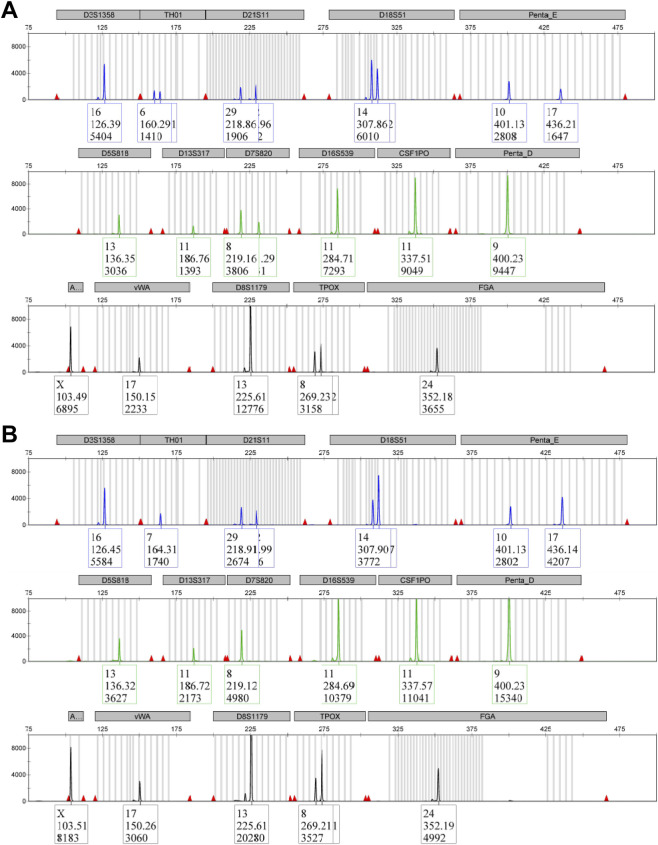
Short tandem repeat (STR) profiling. Electropherograms for parental PC-3 **(A)** and CRISPR/Cas9-derived clone #1 **(B)** are shown. Each peak represents a specific STR locus, including the amelogenin sex marker. All loci matched the known PC-3 reference profile except for the loss of the two-allelic pattern at variant sites D7S820 (8 and 11 repeats in PC-3; 8 repeats in PC-3 LCN2-KO clone #1) and TH01 (6 and 7 in PC-3; 7 in PC-3 LCN2-KO clone #1). This confirms the authenticity and close genetic relationship of clone #1 to the parental cell line.

Short tandem repeat (STR) profiling was conducted to verify the genetic identity and authenticity of both the parental PC-3 cells and the derived clone #1. Analysis of 15 standard STR loci, along with the amelogenin sex marker, showed no discrepancies between the parental PC-3 cell line and the information provided by ATCC, or known references for PC-3 (Masters et al., 2001; van Bokhoven et al., 2003; Lorenzi et al., 2009; Fang et al., 2011; Yu et al., 2015) (Supplementary Table S3; Figure 1). However, clone 1, derived from PC-3 using CRISPR/Cas9 technology, exhibited losses of one STR variant each at variant sites D7S820 and TH01. The parental cell line displayed two signals indicating 8 and 11 repeats at variant site D7S820, and two signals indicating 6 and 7 repeats at variant site TH01, while clone 1 showed only a single signal at these sites. A similar loss at D7S820 was reported in PC-3 cells originating from the National Cell Bank of Iran (Azari et al., 2007). Nevertheless, the genetically modified cell line PC-3 LCN2-KO matched in 14 of the 16 tested markers. Based on the consensus standard, an identity match of 87.5% falls within the range of 80%–100%, confirming the similarity between the parental PC-3 cells and the PC-3 LCN2-KO clone (Korch et al., 2021). However, the absence of variant signals at D7S820 and TH01 may suggest genuine genomic changes in the cell population, such as partial deletions or clonal drift that eliminated these specific alleles.

### 3.3 Microscopic, FACS analysis, and phalloidin stain

**FIGURE 2:**
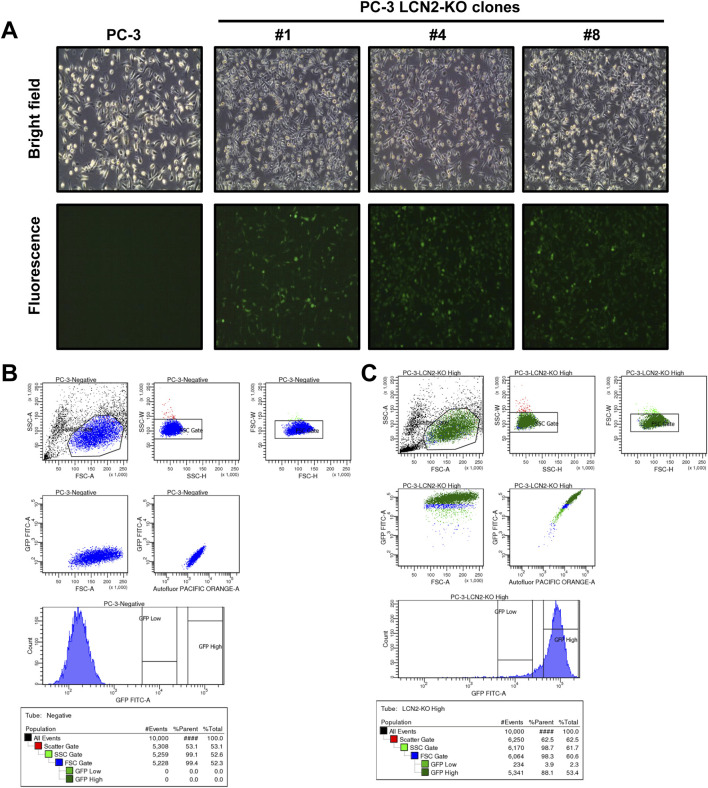
Light microscopic appearance and FACS analysis of PC-3 cells and PC-3 cells lacking *LCN2*. **(A)** Representative bright-field images show the cellular morphology in *LCN2*-deficient PC-3 cells compared to the original PC-3 line. **(B,C)** Flow cytometry was used to evaluate GFP expression from the CRISPR/Cas9 construct. A representative mixture of *LCN2*-KO cells **(C)** display a clearly visible GFP-positive population, while the original parental PC-3 cells **(B)** do not exhibit any GFP expression.

Microscopic examination and FACS analysis were conducted to evaluate cellular morphology and verify GFP fluorescence in CRISPR/Cas9-generated PC-3 LCN2-KO clone #1. Under bright-field illumination, the PC-3 cells lacking *LCN2*, including PC-3 LCN2-KO clone #1 and two other clones, appeared slightly rounded compared to the more elongated morphology of the parental PC-3 cells (Figure 2). To further assess cytoskeletal organization associated with this altered morphology in clones lacking LCN2, we stained filamentous actin (F-actin) using fluorescently labeled phalloidin (Supplementary Figure S1), again demonstrating the altered morphology compared to parental PC-3 cells. This observation confirms our previous finding that the loss of *LCN2* may influence cytoskeletal organization and/or adhesion properties, thereby affecting overall cell shape (Schröder et al., 2022). Moreover, FACS analysis showed a clear GFP signal, originating from the CRISPR/Cas9 clone used to disrupt the *LCN2* gene locus (Schröder et al., 2022), was detected exclusively in the three clones and not in parental PC-3 cells lacking this construct.

### 3.4 Karyotype analysis and spectral karyotype analysis

Cytogenetic analysis of Giemsa-stained metaphases from PC-3 and PC-3 LCN2-KO cells consistently showed a hyperdiploid karyotype, with no diploid cells identified. In approximately 90% of the analyzed metaphases, chromosome numbers ranged between 57 and 61, with a small proportion displaying tetraploidy. Due to the complexity of structural rearrangements that could not be accurately resolved by conventional GTG banding, spectral karyotyping (SKY) was subsequently used.

**FIGURE 3:**
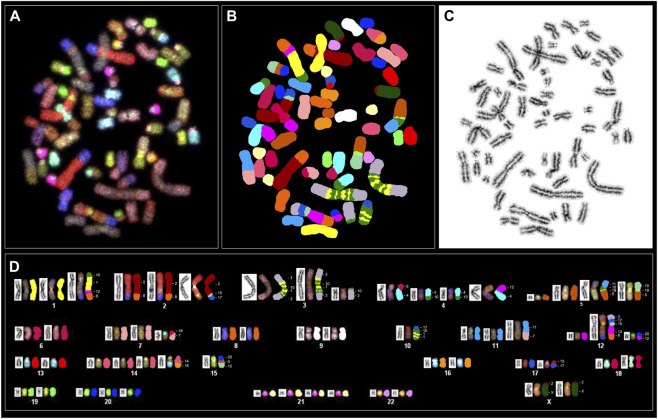
Spectral karyotyping of a human PC-3 metaphase. An RGB image after hybridization with the SKY probe cocktail, containing chromosome-specific fluorescent probes in distinct colors **(A)** is shown alongside the spectrally classified pseudo-colored image **(B)** the corresponding inverted DAPI-stained image **(C)** and the karyotype. The karyotype below **(D)** presents spectrally classified pseudo-colored chromosomes (*right*), their inverted DAPI-stained counterparts (*left*), and the RGB images (*middle*). Chromosome segments involved in the rearrangements are labeled with their corresponding numeric and letter designations. The rearranged chromosomes are classified according to the presence of the largest segment from the respective chromosomes. Based on SKY in the majority of metaphases, the PC-3 karyotype can be designated as: <57-61>,XX,-Y,+1,der(1)t(1;8;10;12),+2,der(2)t(2;8)x2,der(2)t(2;15;17),+3,der(3)t(1;3;10)x2,der(3)t(3;10),+4,der(4)t(4;6),der(4)t(4;10)x2,der(4)t(4;12),+5,der(5)t(5;10;11;19),der(5)t(5;15;19),+7,der(7)t(7;18),-10,der(10)t(1;10;17),+11,der(11)t(7;11),+12,der(12)t(3;8;12;15;17),der(12)t(12;20),+14,der(14)t(14;16),-15,der(15)t(5;15;20),der(17)t(13;17),+20,+21,der(X)t(2;X)x2. (+) denotes the gain or presence of extra copies of a specific chromosome, whereas (−) denotes the loss or deletion of a specific chromosome.

**FIGURE 4:**
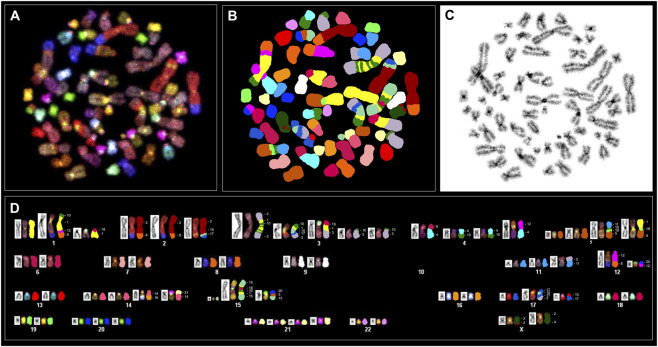
Spectral karyotyping of a human PC-3 LCN2-KO metaphase. An RGB image after hybridization with the SKY probe cocktail, containing chromosome-specific fluorescent probes in distinct colors **(A)** is shown alongside the spectrally classified pseudo-colored image **(B)** the corresponding inverted DAPI-stained image **(C)** and the karyotype. The karyotype below **(D)** presents spectrally classified pseudo-colored chromosomes (*right*), their inverted DAPI-stained counterparts (*left*), and the RGB images (*middle*). Chromosome segments involved in the rearrangements are labeled with their corresponding numeric and letter designations. Translocated chromosomes were classified according to the chromosome from which the larger segment originated. Based on SKY in the majority of metaphases, the PC-3 LCN2-KO karyotype can be described as:<57-60>,XX,+Y, +1,der(1)t(1;8;10;12),der(1)t(1;18),+2,der(2)t(2;8)x2,der(2)t(2;15;17), +3,der(3)t(1;3;10),der(3)t(1;3;10;15;17),der(3)t(1;3;18),der(3)t(3;10)x2,+4,der(4)t(4;6),der(4)t(4;10)x2,der(4)t(4;12),+5,der(5)t(5;10;11;19),der(5)t(1;5;19),+10,+11,der(11)t(3;11),der(12)t(8;12),der(12)t(12;20),+14,der(14)t(14;16),der(14)t(14;21),+15,der(15)t(1;10;15;18),der(15)t(5;15;20), +17,der(17)t(3;15;17),der(17)t(13;17),+20,+21,der(X)t(2;X)x2. (+) denotes the gain or presence of extra copies of a specific chromosome, whereas (−) denotes the loss or deletion of a specific chromosome.

SKY analysis allowed for the simultaneous identification of all rearranged chromosomes, providing an overview of the chromosome segments involved in rearrangements across the analyzed metaphases. The large chromosomes HSA1, HSA2, HSA3, HSA4 and HSA5 were most frequently affected in both PC-3 and the derived PC-3 LCN2-KO cells, often present in multiple copies and exhibiting multiple translocations. Additionally, chromosomes HSA10, HSA11, HSA12, HSA14, HSA15, HSA17, and X also displayed rearrangements. Specific translocations in both karyotypes can be seen in Figures 3, 4 as well as the accompanying Supplementary Table S4.

Most of these rearrangements were recurrent, appearing in the majority of metaphases analyzed, and were unbalanced, suggesting potential regional copy number alterations. In addition to these changes, chromosomes 20 and 21 were present in multiple copies in both cell lines. Consistent with previous reports (Pan et al., 1999), the Y chromosome was absent in PC-3 cells and similarly undetectable in PC-3 LCN2-KO cells. Importantly, chromosome 9, which contains the targeted *LCN2* gene for knockdown, showed no rearrangement.

Both cell lines exhibit highly complex karyotypic rearrangements, with each rearranged chromosome comprising segments derived from multiple distinct chromosomes. In PC-3 cells (Figure 3), chromosome 1 (HSA1) is present in three copies, two of which are intact, while the third is rearranged and contains smaller segments originating from HSA8, HSA10, and HSA12. Additional HSA1 fragments are also detected on chromosomes HSA3 and HSA10. In contrast, in PC-3 LCN2-KO cells (Figure 4), HSA1 likewise occurs in three copies; however, only one copy remains intact, whereas the other two are extensively rearranged. Moreover, further HSA1-derived segments are observed on chromosomes HSA3, HSA5, and HSA15.

**FIGURE 5:**
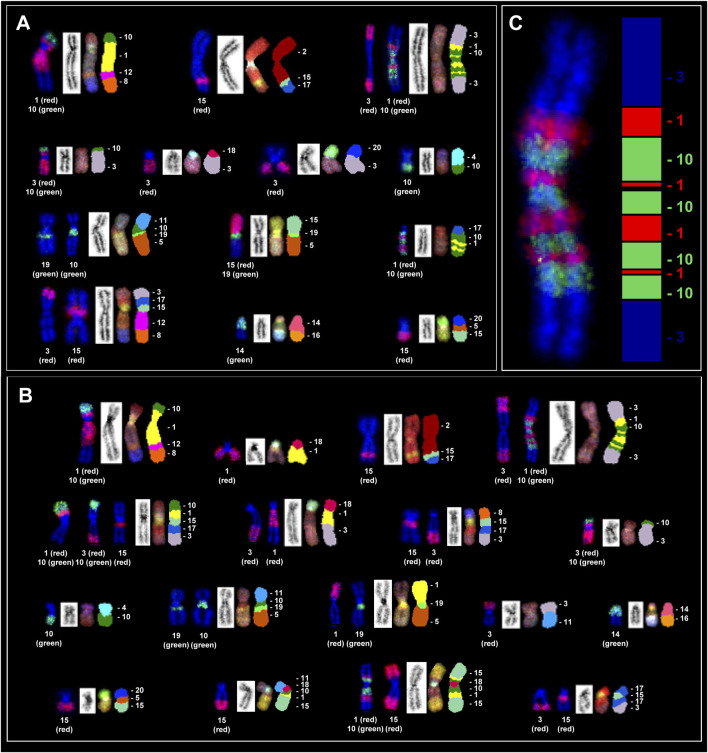
Validation of SKY hybridization patterns by FISH using individual chromosome paints in both cell lines. Single-chromosome FISH results for **(A)** PC-3 and **(B)** PC-3 LCN2-KO are shown on the left, with the corresponding SKY images depicting the rearranged chromosomes on the right. **(C)** FISH with chromosome paints for chromosomes 1 and 10 in high resolution revealing interspersed segments within the rearranged complex chromosome 3, as shown in the SKY images. The adjacent schematic depicts the arrangement of each chromosomal segment (chromosome 1, colored in red, chromosome in DAPI stain in blue, and chromosome 10 in green).

The pattern of rearrangements involving chromosome HSA3 is strikingly different between the two cell lines, with none of the chromosomes remaining intact. In PC-3 cells, all three copies of HSA3 contain translocated segments from HSA10. Additionally, in two identical large, complex rearranged chromosomes, segments from HSA1 and HSA10 are interspersed along both the short and long arms around the centromere. FISH experiments with single chromosome paints were carried out in metaphases of both lines to validate complex translocation as well as the SKY hybridization pattern (Figure 5).

In the knockout PC-3 LCN2-KO cells, HSA3 is present in more than three copies, most of which contain segments from HSA10. The large, complex translocated chromosome that comprises material from HSA3, HSA1, and HSA10 and is present in two copies in PC-3 cells is reduced to a single copy in the knockout cells. Additionally, four smaller rearranged copies of chromosome 3 were observed: one carrying segments from HSA1 and HSA18; and two smaller copies including segments from HSA10.

The rearrangement patterns of most HSA5 copies were largely similar between the two cell lines. However, one rearranged copy in PC-3 LCN2-KO included a segment derived from HSA1, whereas the corresponding region in PC-3 contained sequences from HSA15. Notably, a single rearranged HSA10 in PC-3 harbored two segments from HSA1 and one from HSA17, whereas in PC-3 LCN2-KO, both copies of HSA10 were involved in translocations across nine different rearranged chromosomes. These observations suggest that *LCN2* knockout is associated with additional chromosomal rearrangements, particularly involving HSA10 and HSA5, indicating potential changes in genome stability in the knockout line.

Additional differences are observed in chromosomes HSA14, HSA15, and HSA17, which display specific rearrangements in PC-3 LCN2-KO. For HSA12, PC-3 cells exhibit segments from HSA3, HSA12, HSA15, and HSA17, whereas the knockout cells show only a single translocation between HSA8 and HSA12. A more detailed comparison of the rearrangement patterns between these two cell lines is highlighted in Table 1.

**TABLE 1:** Frequency of translocations present in PC-3 and PC-3 LCN2-KO.

Frequency of translocations	PC-3	PC-3 LCN2-KO	Common
Translocations with lower frequency in 20 metaphases (7-11x)	t (17; 10; 1;10; 1;10) t (3; 17; 15; 1;10) t (3; 17; 15; 17) t (8; 12)	t (7; 18)	—
Translocations with higher frequency in 20 metaphases (12-16x)	t (7; 11)	t (3; 1;18) t (15; 1;10; 18; 10; 1;15) t (8; 12; 1;10) t (5; 10; 11) t (14; 16)	—
Major translocations (17-20x)	t (5; 19; 15) t (8; 12; 1;10) t (5; 19; 10; 11) t (14; 16)	t (5; 19; 1) t (11; 3) t (3; 17; 15; 1;10) t (3; 17; 15; 17) t (8; 12)	t (8; 2) t (17; 15; 2) t (3; 10; 1;10; 1;10; 1;3) t (4; 6) t (4; 10) t (4; 12) t (12; 20) t (15; 5;20) t (17; 13) t (X; 2)

SKY analysis of PC-3 and PC-3 LCN2-KO cell lines revealed 24 structurally rearranged chromosomes in the majority of 20 metaphases, with 17 aberrations (71%) shared between the two lines, indicating a largely conserved karyotypic background. Notably, distinct translocations were unique to each line: t (3; 18), t (5; 19; 1), t (11; 3), and t (15; 1;10; 18; 10; 1;15) were exclusive to PC-3 LCN2-KO, whereas t (5; 19; 15), t (17; 10; 1;10; 10), and t (7; 11) were detected only in PC-3.

Most copies of HSA5 exhibited similar rearrangement patterns in both lines; however, one rearranged HSA5 in PC-3 LCN2-KO included a segment from HSA1, contrasting with HSA15 in the parental line. Strikingly, while a single HSA10 in PC-3 carried two segments from HSA1 and one from HSA17, both HSA10 copies in PC-3 LCN2-KO were redistributed across nine different rearranged chromosomes. These differences suggest that *LCN2* knockout contributes to additional, non-random chromosomal rearrangements, particularly involving HSA10 and HSA5, reflecting altered genome stability.

Although recurrent rearrangements are highlighted in the table, additional sporadic aberrations were also observed among the 20 metaphases analyzed. In total, PC-3 LCN2-KO cells displayed a higher total number of chromosomal aberrations (69) compared to the parental PC-3 line (59) (Supplementary Table S5). Furthermore, a comparison of the frequencies of specific chromosomes involved in rearrangements among the 20 analyzed metaphases revealed that 11 different chromosomes were more frequently rearranged in PC-3 LCN2-KO cells, compared to only three chromosomes in the parental PC-3 line (Supplementary Table S6). This finding suggests that the knockout cells may have an increased propensity for chromosomal instability relative to the parental PC-3 cells.

Analysis of detectable translocated segments relative to the normal diploid complement revealed a significant overall chromosomal gain in both lines (Table 2). However, SKY analysis is unable to detect losses. Additionally, DAPI-stained interphase nuclei consistently displayed micronucleus formation, either attached to or adjacent to the main nucleus. The frequency of micronuclei was significantly higher in PC-3 LCN2-KO than in PC-3 (Supplementary Figure S2), indicating a continuous ongoing elimination of chromosomal fragments before metaphase and emphasizing enhanced genomic instability in the knockout cells.

**TABLE 2:** Overall chromosomal gains and losses based on the SKY analysis, including segments of individual chromosomes identified by their hybridization signatures within structurally rearranged chromosomes.

PC-3				
Chromosome	Copy number (modal) (See Figure 3)	Loss or gain observed in PC-3^a^ (# cells/total)		Interpretation
1	3–4^e^	Gain: No gain, no loss: Loss:	9/10 1/10 0/10	Gain
2	3^d^ ^,^ ^f^ ^,^ ^g^	Gain: No gain, no loss: Loss:	10/10 0/10 0/10	Gain
3	2^f^–3^f^ ^,^ ^g^	Gain: No gain, no loss: Loss:	8/10 1/10 1/10	Gain
4	2^d^ ^,^ ^g^	Gain: No gain, no loss: Loss:	0/10 9/10 1/10	Unchanged
5	2,5^d^ ^,^ ^f^ ^,^ ^g^	Gain: No gain, no loss: Loss:	10/10 0/10 0/10	Gain
6	2,5^d^	Gain: No gain, no loss: Loss:	9/10 1/10 0/10	Gain
7	3^f^	Gain: No gain, no loss: Loss:	10/10 0/10 0/10	Gain
8	4^d^	Gain: No gain, no loss: Loss:	10/10 0/10 0/10	Gain
9	2	Gain: No gain, no loss: Loss:	0/10 10/10 0/10	Unchanged
10^b^	2,5^e^ ^,^ ^f^ ^,^ ^g^	Gain: No gain, no loss: Loss:	10/10 0/10 0/10	Gain
11	3,5^d^ ^,^ ^f^	Gain: No gain, no loss: Loss:	10/10 0/10 0/10	Gain
12	2^f^ ^,^ ^g^	Gain: No gain, no loss: Loss:	2/10 7/10 1/10	Unchanged
13	∼2^f^	Gain: No gain, no loss: Loss:	8/10 0/10 2/10	Gain
14	3,5^f^	Gain: No gain, no loss: Loss:	10/10 0/10 0/10	Gain
15	∼2^d^ ^,^ ^f^ ^,^ ^g^	Gain: No gain, no loss: Loss:	8/10 2/10 0/10	Gain
16	2,5^d^	Gain: No gain, no loss: Loss:	10/10 0/10 0/10	Gain
17	3^d^ ^,^ ^f^	Gain: No gain, no loss: Loss:	7/10 2/10 1/10	Gain
18	3^d^	Gain: No gain, no loss: Loss:	9/10 0/10 1/10	Gain
19	∼2^f^	Gain: No gain, no loss: Loss:	10/10 0/10 0/10	Gain
20	4^d^ ^,^ ^f^	Gain: No gain, no loss: Loss:	10/10 0/10 0/10	Gain
21	4^d^	Gain: No gain, no loss: Loss:	10/10 0/10 0/10	Gain
22	2	Gain: No gain, no loss: Loss:	0/10 9/10 1/10	Unchanged
X^c^	2^g^	Gain: Loss:	10/10 10/10	Gain and loss
Y	0	No Y-chromosome	10/10	—

PC-3 LCN2-KO				
Chromosome	Copy number (modal) (See Figure 4)	Loss or gain observed in PC-3 LCN2-KO^a^(# cells/total)		Interpretation
1	3,5***^,f^	Gain: No gain, no loss: Loss:	10/10 0/10 0/10	Gain
2	3^d,f,g^	Gain: No gain, no loss: Loss:	10/10 0/10 0/10	Gain
3	∼3***^,f,g^	Gain: No gain, no loss: Loss:	10/10 0/10 0/10	Gain
4	2^d,g^	Gain: No gain, no loss: Loss:	8/10 2/10 0/10	Gain
5	2–3^d,f,g^	Gain: No gain, no loss: Loss:	9/10 1/10 0/10	Gain
6	2,5^d^	Gain: No gain, no loss: Loss:	10/10 0/10 0/10	Gain
7	2^f^	Gain: No gain, no loss: Loss:	9/10 1/10 0/10	Gain
8	4^d^	Gain: No gain, no loss: Loss:	10/10 0/10 0/10	Gain
9	2	Gain: No gain, no loss: Loss:	1/10 9/10 0/10	Unchanged
10^b^	4^e,f,g^	Gain: No gain, no loss: Loss:	9/10 1/10 0/10	Gain
11	3^d,f^	Gain: No gain, no loss: Loss:	10/10 0/10 0/10	Gain
12	1,5^f,g^	Gain: No gain, no loss: Loss:	4/10 6/10 0/10	Unchanged
13	∼2^f^	Gain: No gain, no loss: Loss:	810 2/10 0/10	Gain
14	3,5^f^	Gain: No gain, no loss: Loss:	10/10 0/10 0/10	Gain
15	3,5^d,f,g^	Gain: No gain, no loss: Loss:	9/10 1/10 0/10	Gain
16	2,5^d^	Gain: No gain, no loss: Loss:	10/10 0/10 0/10	Gain
17	3,5^d,f^	Gain: No gain, no loss: Loss:	6/10 3/10 1/10	Gain
18	3^d,f^	Gain: No gain, no loss: Loss:	6/10 4/10 0/10	Gain
19	∼2^f^	Gain: No gain, no loss: Loss:	8/10 2/10 0/10	Gain
20	4^d,f^	Gain: No gain, no loss: Loss:	10/10 0/10 0/10	Gain
21	6^d^	Gain: No gain, no loss: Loss:	10/10 0/10 0/10	Gain
22	2	Gain: No gain, no loss: Loss:	1/10 9/10 0/10	Unchanged
X^c^	2^g^	Gain: Loss:	10/10 10/10	Gain and loss
Y	0	No Y-chromosome	10/10	—

^a^ Compared to two normal copies.

^b^ Different segments translocated to other chromosomes.

^c^ Gain of one smaller metacentric X-chromosome.

^d^ Numerical (whole chromosome, p-arm, q-arm).

^e^ Complex rearranged segments.

^f^ Translocated segments and insertions.

^g^ No intact copy; SKY, hybridization does not allow detection of chromosomal loss.

^a^Compared to two normal copies.

^b^Different segments translocated to other chromosomes.

^c^Gain of one smaller metacentric X-chromosome.

^d^Numerical (whole chromosome, p-arm, q-arm).

^e^Complex rearranged segments.

^f^translocated segments and insertions.

^g^No intact copy; SKY, hybridization does not allow detection of chromosomal loss.

*** d,e.

### 3.5 Comparative next-generation sequencing in PC-3 and PC-3 LCN2-KO

Next, we conducted next-generation sequencing (NGS) on both the original PC-3 cells (Supplementary Table S7) and PC-3 LCN2-KO clone #1 (Supplementary Table S8) to identify changes in gene expression associated with the CRISPR/Cas9-mediated *LCN2* knockout. This analysis allowed us to thoroughly compare mRNA profiles, enabling us to identify genes that were either upregulated or downregulated due to the absence of *LCN2* in clone #1.

As a control, our NGS data indicated that the expression of *LCN2* in clone #1 was absent compared to that in the parental cell line PC-3 (Supplementary Table S9). Additionally, we confirmed previous findings that PC-3 cells did not express p53 (*TP53*) and the androgen receptor (*AR*). Furthermore, the expression of PTEN was relatively low.

**FIGURE 6:**
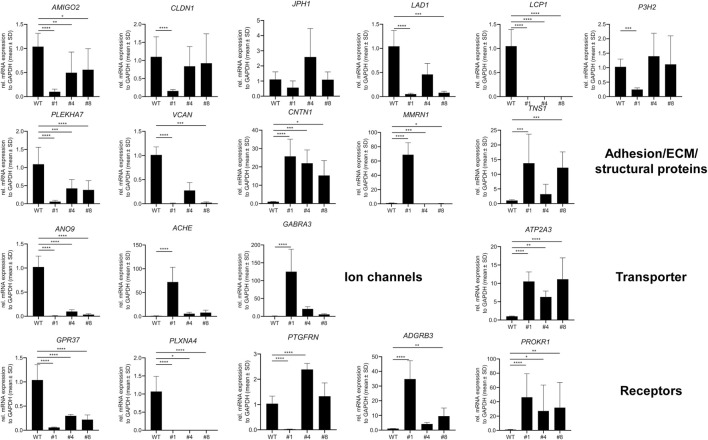
Confirmation of next-generation data by RT-qPCR of genes encoding adhesion/extracellular matrix proteins, ion channels, transporters, receptors. Genes that exhibited altered expression in next-generation sequencing were analyzed using RT-qPCR in PC-3, clone #1, and other independent clones (clones #4 and #8) with disrupted *LCN2* gene. Statistical significance: *p* > 0.05 (ns), *p* < 0.05 (*), *p* < 0.01 (**), *p* < 0.001 (***), *p* < 0.0001 (****).

**FIGURE 7:**
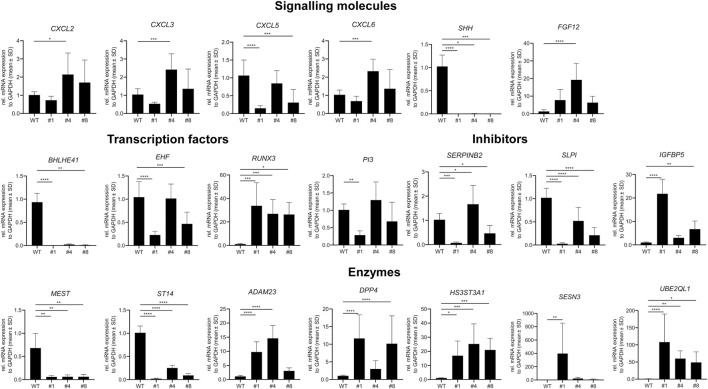
Confirmation of next-generation data by RT-qPCR of genes encoding signaling molecules, transcription factors, inhibitors, and enzymes. Genes that showed altered expression in next-generation sequencing were tested by RT-qPCR in PC-3, clone 1, and additional independent clones (clones #4 and #8) with disrupted *LCN2* gene. Statistical significance: *p* > 0.05 (ns), *p* < 0.05 (*), *p* < 0.01 (**), *p* < 0.001 (***), *p* < 0.0001 (****).

To further validate the reliability of our NGS data, we conducted RT-qPCR to analyze the expression of genes that showed significant downregulation (*AMIGO2*, *CLDN1*, *JPH1*, *LAD1*, *LCP1*, *P3H2*, *PLEKHA7*, *VCAN*, *ANO9*, *ATP2A3*, *CXCL2*, *CXCL3*, *CXCL6*, *SHH*, *BHLHE41*, *EHF*, *RUNX3*, *PI3*, *SERPINB2*, *SLPI*, *MEST*, and *ST14*) or upregulation (*CNTN1*, *MMRN1*, *TNS1*, *ACHE*, *GABRA3*, *GPR37*, *PLXNA4*, *PTGFRN*, *ADGRB3*, *PROKR1*, *FGF12*, *IGFBP5*, *ADAM23*, *DPP4*, *HS3ST3A1*, *SESN3*, and *UBE2QL1*) in clone #1 compared to parental PC-3 cells. These genes encode proteins relevant for adhesion, extracellular matrix synthesis, structural proteins, ion channels, transporters, and receptors (Figure 6), or genes encoding signaling molecules, transcription factors, inhibitors, or enzymes (Figure 7). Additionally, we included two other independent PC-3 clones lacking *LCN2* (clones #4 and #8) in this analysis to determine if the observed differential expression is specific to clone #1 or a more general observation associated with the loss of *LCN2* in PC-3 cells.

This analysis confirmed our NGS data, indicating significant differences in gene expression in PC-3 compared to clone #1, with only the *JPH1* gene showing no differential expression. Among the genes tested, *AMIGO2*, *LCP1*, *PLEKHA7*, *ANO9*, *GPR37*, *PLXNA4*, *SHH*, *SLPI*, *MEST*, and *ST14* were significantly downregulated in all clones lacking *LCN2*, while *CNTN1*, *ATP2A3*, *PROKR1*, *RUNX3*, and *HS3ST3A1* were significantly upregulated, suggesting that these genes are directly or indirectly influenced in their expression by *LCN2*.

**FIGURE 8:**
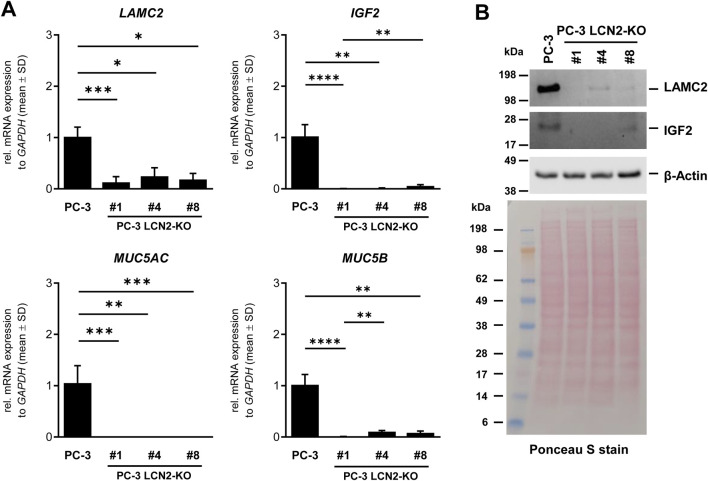
Expression analysis by RT-qPCR and Western blot analysis. **(A)** mRNA expression of *LAMC2*, *IGF2*, *MUC5AC*, and *MUC5B* was analyzed by RT-qPCR, showing that these genes are significantly lower expressed in PC-3 clones lacking *LCN2* compared to parental PC-3 cells. **(B)** Western blot analysis of LAMC2 and IGF2 in PC-3 cells and derivatives lacking *LCN2*. To confirm equal protein loading the membrane was stained with Ponceau S and further probed with an antibody specific for β-actin. Statistical significance: *p* > 0.05 (ns), *p* < 0.05 (*), *p* < 0.01 (**), *p* < 0.001 (***), *p* < 0.0001 (****).

In another set of experiments, we analyzed the expression of *LAMC2*, *IGF2*, *MUC5AC*, and *MUC5B* by RT-qPCR (Figure 8A). Consistent with our NGS data (Table 3), these genes showed significant suppression in clone #1 and even more so in the other two clones lacking *LCN2*. To confirm that the lower expression in these clones is also present at the protein level, we conducted Western blot analysis for the expression of LAMC2 and IGF2 (Figure 8B), demonstrating that the reduced mRNA levels of these genes are reflected at the protein level as well. In this analysis, we focused our protein-level validation on LAMC2 and IGF2 because they showed strong and consistent differential expression and represent two biologically important axes affected by LCN2 loss-extracellular matrix remodeling and tumor progression (LAMC2) versus growth factor signaling and proliferation (IGF2). These candidates were also selected due to the availability of well-validated antibodies suitable for robust Western blot analysis, allowing us to confirm the transcriptomic changes at the protein level. Thus, LAMC2 and IGF2 should be regarded as illustrative markers of broader pathway alterations rather than an exhaustive validation panel, and a more comprehensive, systematic protein-level characterization of additional targets remains an important task for future studies using this LCN2-deficient model.

**TABLE 3:** Expression data on genes previously suggested to be deleted in PC-3 cells.

Chromosome	Gene symbol	Description	Expression (based on NGS) TPM PC-3/TPM PC-3 LCN2-KO (clone #1)^a^
Y	*DDX3Y*	DEAD-box helicase 3 Y-linked	0.125989/0
	*NLGN4Y*	Neuroligin 4 Y-linked	0.100862/0
	*TMSB4Y*	Thymosin beta 4 Y-linked	0/0
	*TSPY1*	Testis specific protein Y-linked 16, pseudogene	0/0
	*TTTY13*	Testis expressed transcript, Y-linked 13 (noncoding)	NN
	*TTTY15*	Testis expressed transcript, Y-linked 15 (noncoding)	NN
	*USP9Y*	Ubiquitin specific peptidase 9 Y-linked	3.52108/3.58957 0.717954/0 0.483014/0.215386 0/0.224663
	*UTY*	Ubiquitously transcribed tetratricopeptide repeat containing, Y-linked	0/0.123914
5	*CDH18*	Cadherin 18	0.158528/0.403599 0/1.15075
	*CTNNA1*	Catenin alpha 1	14.6452/22.7125 5.62364/2.15075 1.12536/0.944553
	*LRRTM2*	Leucine rich repeat transmembrane neuronal 2	0/0
	*SIL1*	SIL1 nucleotide exchange factor	8.10422/1.23822 7.54798/0 4.34778/5.67058 1.41299/0.971329 0/0.598293 0/2.93041 0/0.00000930553 0/1.72796 0/0.532011
10	*DYDC1*	DPY30 domain containing 1	0/0
	*DYDC2*	DPY30 domain containing 2	1.38573/0 0.000270945/1.45388
	*FAM213A* *(PRXL2A*)	Family with sequence similarity 213 member A (peroxiredoxin like 2A)	1.24251/1.46849
	*LIPJ*	Lipase family member J	0/0
	*MAT1A*	Methionine adenosyltransferase 1A	0/0
	*PTEN*	Phosphatase and tensin homolog	3.7314/3.58018 3.37523/2.7729 3.37523/2.7729 1.70385/1.42223 1.08974/0.597133 0.298957/0.131053 0.285352/0.176785 0.285352/0.176785 0.131945/0.175493 0/0.172285
	*RNLS*	Renalase, FAD dependent amine oxidase	0.166315/0.141014
	*SH2D4B*	SH2 domain containing 4B	0/0
	*TSPAN14*	Tetraspanin 14	0.203657/0 0.0994184/0.114508 0.000000279281/0

Chromosome	Gene symbol	Description	Expression (based on NGS) TPM PC-3/TPM PC-3 LCN2-KO (clone #1)^a^
17	*ATP6V0A1*	ATPase H+ transporting V0 subunit a1	0.763929/0 0.132375/0.159541 0.1195/0.136854 0.0689719/0 0.000244866/0.000248664 0/0.000000359557 0/0.918461 0/0.256181
	*DHX58*	DExH-box helicase 58	0/0
	*GHDC*	GH3 domain containing	0/0.366065
	*HCRT*	Hypocretin neuropeptide precursor	0/0
	*HSPB9*	heat shock protein family B (small) member 9	0/0
	*KAT2A*	Lysine acetyltransferase 2A	0/0
	*KCNH4*	Potassium voltage-gated channel subfamily H member 4	0/0
	*MIR548AT*	microRNA 548 at	NN
	*PTRF* *(CAVIN1)*	Phosphatase and tensin homolog (caveolae associated protein 1)	0.31948/0.399934
	*RAB5C*	RAB5C, member RAS oncogene family	2.19793/0.619878 1.50522/0.238382
	*STAT3*	Signal transducer and activator of transcription 3	1.33698/1.12365 0.828543/0 0.112749/0
	*STAT5A*	Signal transducer and activator of transcription 5A	0/0
	*STAT5B*	Signal transducer and activator of transcription 5B	1.37025/0.671039 1.31239/0 0/0.558876
19	*CIC*	Capicua transcriptional repressor	5.37448/6.06077 0/0.508768
	*TMEM145*	Transmembrane protein 145	21.6361/24.6839 0.305262/0.747633
	*PAFAH1B3*	Platelet activating factor acetylhydrolase 1b catalytic subunit 3	0/0
	*PRR19*	Proline rich 19	0.827967/0.874992
20	*SIRPB1*	Signal regulatory protein beta 1	15.6587/1.94538 13.58/1.45743 12.8338/0 7.07159/1.55357 1.04202/0.318064 0.70592/0 0.499951/0 0.465022/0

^a^ In this row each line corresponds to a specific transcript of the listed gene. For identity of specific transcripts of listed genes refer to Supplementary Tables S7 and S8. NN, not analyzed in our NGS, expression analysis.

## 4 Discussion

In this study, we focused on the functional and genomic characterization of a CRISPR/Cas9-derived PC-3 subclone (clone #1) lacking *LCN2* and compared it to the parental PC-3 cell line. Our findings highlight both the value of PC-3 as a well-characterized model system for advanced prostate cancer and the unique features that emerge following disruption of *LCN2*. Taken together, they provide new insights into how *LCN2* regulates important molecular pathways in prostate cancer cells and illustrate ways in which *LCN2*-deficient PC-3 cells can be exploited to dissect disease mechanisms and test therapeutic strategies.

A first difference between parental PC-3 and PC-3 LCN2-KO is the partial loss of STR markers (e.g., at D7S820 and TH01), hinting at minor genomic alterations that arose during or after CRISPR/Cas9 editing. Notably, these changes did not compromise the fundamental identity of the new subclone relative to parental PC-3, as most markers and major structural rearrangements were largely conserved. Indeed, both PC-3 and PC-3 LCN2-KO display the hallmark aneuploidy and chromosomal translocations typical of PC-3 cells, underscoring their common genetic backbone.

SKY analysis of PC-3 and PC-3 LCN2-KO cells revealed highly complex and heterogeneous karyotypes, a hallmark of prostate cancer cell lines. The high incidence of complex rearrangements spanning multiple chromosomes observed in the present study may be explained by chromoplexy, a phenomenon initially identified in prostate cancer by Baca et al. and more recently elaborated by Pellestor and colleagues (Baca et al., 2013; Pellestor et al., 2025). The TMPRSS2–ERG fusion on chromosome 21 is a well-known driver in prostate cancer and is often associated with chromoplexy. Although this fusion was not directly assessed in the present study, HSA21 was observed in multiple copies in both cell lines, indicating genomic instability. Genes commonly disrupted by chromoplectic events PTEN, FOXA1, and SPOP are located on HSA10, HSA14, and HSA17, respectively, all of which exhibited extensive rearrangements in PC-3 LCN2-KO compared with parental PC-3 cells. These findings suggest that chromoplexy-like mechanisms may contribute to the complex karyotypic alterations in PC-3 LCN2-KO, potentially impacting key tumor suppressor genes involved in prostate cancer.

Both lines displayed a hyperdiploid chromosome number, consistent with previous descriptions of PC-3 cells as genomically unstable and aneuploid (Pan et al., 1999). Some of the complex translocations identified in this study are novel and have not been reported previously, reflecting the inherent tendency of this cell line to continuously acquire new rearrangements and evolve its karyotype over time. Consequently, future experiments using these cells should incorporate karyotype analysis as a key parameter to account for cellular heterogeneity.

SKY analysis confirmed that large chromosomes, particularly HSA1, HSA2, HSA3, HSA4, and HSA5, were the most frequently involved in structural rearrangements in both lines. These chromosomes were often present in multiple copies and exhibited complex, unbalanced translocations, indicative of widespread copy number alterations. Additional recurrent involvement of chromosomes HSA10, HSA11, HSA12, HSA14, HSA15, HSA17, and X further highlights the extensive genomic reorganization within these cells. Chromosome 9, which harbors the *LCN2* gene, showed no structural rearrangements, confirming that the knockout did not introduce large-scale changes at the targeted locus; rather, all observed rearrangements are off-target.

Comparative SKY mapping between PC-3 and PC-3 LCN2-KO cells revealed that 71% of the identified rearrangements were shared, indicating a largely conserved chromosomal background inherited from the parental line. However, the knockout cells exhibited additional unique and complex translocations, notably t (3; 18), t (5; 19; 1), and t (15; 1;10; 18; 10; 1;15), suggesting that LCN2 deletion may have downstream effects on chromosome stability. The increased structural complexity involving HSA10 and HSA5 in PC-3 LCN2-KO cells is particularly striking, with these chromosomes incorporated into a greater number of rearranged structures compared to the parental line. Given that loss of the tumor suppressor *PTEN*, located on HSA10, is known to induce extreme chromosomal instability (Pei et al., 2022), the redistribution of HSA10 across nine rearranged chromosomes may point to a potential role of *LCN2* in maintaining genomic integrity.

Overall, the total number of aberrations was higher in PC-3 LCN2-KO (69) than in PC-3 (59), with more chromosomes affected per metaphase, indicating enhanced chromosomal instability in the knockout line. The presence of numerous micronuclei in interphase nuclei of PC-3 LCN2-KO cells further supports this conclusion, reflecting ongoing chromosomal breakage and missegregation. These findings are consistent with a role for *LCN2* in cellular stress responses and genome maintenance, where its loss may compromise DNA damage repair pathways or mitotic checkpoint control, promoting structural chromosome instability and complex rearrangements.

It should be emphasized that, in addition to *PTEN* loss, PC-3 cells display abnormalities in the tumor suppressor *TP53*. Chromosome HSA17, which contains *TP53*, is organized differently between the two cell lines. In PC-3 LCN2-KO cells, HSA17 is present in three copies, with one copy exhibiting a complex rearrangement absent in the parental PC-3 cells. Considering that LCN2 regulates intracellular iron levels, affecting cell proliferation, metabolism, and oxidative stress, the combined effect of *LCN2* loss, *PTEN* deficiency, and *TP53* abnormalities likely contributes to the increased frequency of chromosomal aberration observed in the knockout cells.

Taken together, these results indicate that while PC-3 and PC-3 LCN2-KO cells share a broadly conserved but abnormal karyotype, the *LCN2* knockout line exhibits additional non-random chromosomal alterations and enhanced cytogenetic instability. These alterations likely contribute to the phenotypic differences observed between the two lines and may reflect an adaptive response to the loss of *LCN2*-mediated genomic homeostasis.

Previous data, collected 1 year after the establishment of the PC-3 cell line, indicated that PC-3 cells have a near-triploid karyotype with 11 marker chromosomes and are missing chromosomes 2, 3, 5, and 15. Several segments of the missing chromosomes have combined with other chromosomal segments to form these markers (Ohnuki et al., 1980). In a subsequent study, Pan and colleagues identified genomic aberrations involving 14 chromosomes and 16 different translocated chromosomes, including t(10; 1;10), t(2; 8), t(2; 15; 17), t(3; 10), t(4; 6), t(4; 10), t(4; 10; 15), t(5; 15), t(8; 14; 11), t(3; 10; 3), t(5; 10; 11), t(8; 12), t(4; 12), t(14; 15), t(3; 10; 15; 14; 16), and t(3; 17; 15; 17) (Pan et al., 1999). The authors also observed two subpopulations within PC-3 cells, one with 58 chromosomes and the other with 113 chromosomes, suggesting a tendency for PC-3 cells to form subpopulations with a duplicated genome during cell culture (Pan et al., 1999). We were able to confirm the presence of most of these marker chromosomes, with the t (3; 10; 1;10; 1;10; 1;3) marker being the most characteristic for PC-3 cells. Furthermore, we identified a PC-3 LCN2-KO clone #1 cell exhibiting a karyotype of “101, XXXX”, which supports the notion that PC-3 cells are capable of generating subpopulations with a duplicated genome.

Whole-genome sequencing revealed that large regions of the Y chromosome including eight genes (*DDX3Y*, *NLGN4Y*, *TMSB4Y*, *TSPY1*, *TTTY13*, *TTTY15*, *USP9Y*, and *UTY*) were deleted in PC-3 cells (Seim et al., 2017). Similarly, several genes on chromosome 5 (*CDH18*, *CTNNA1*, *LRRTM2*, and *SIL1*), chromosome 10 (*DYDC1*, *DYDC2*, *FAM213A*, *LIPJ*, *MAT1A*, *PTEN*, *RNLS*, *SH2D4B*, and *TSPAN14*), chromosome 17 (*ATP6V0A1*, *DHX58*, *GHDC, HCRT*, *HSPB9*, *KAT2A*, *KCNH4*, *MIR548AT*, *PTRF*, *RAB5C*, *STAT3*, *STAT5A*, and *STAT5B*), chromosome 19 (*CIC*, *PAFAH1B3*, *PRR19*, and *TMEM145*), and chromosome 20 (*SIRPB1*) have been reported as deleted in PC-3 cells in previous studies (Liu et al., 2008; Krohn et al., 2012; The Cancer Genome Atlas Research Network, 2015; Ibeawuchi et al., 2015; Seim et al., 2017). Our unbiased NGS analysis confirmed the absence or negligible expression of these genes (except *USP9Y*, *CTNNA1, SIL1, PTEN, RAB5C, CIC, TMEM145,* and *SIRPB1*) in PC-3 cells (Table 3). These findings indicate that PC-3 cells harbor extensive genomic deletions affecting multiple chromosomes and genes, several of which have established roles in cell signaling, metabolism, and tumor suppression. In some cases these regions contain tumor suppressor genes that are associated with prostate cancer when lost or mutated (Dong et al., 2008).

By confirming the absence or significantly reduced expression of these genes, our unbiased NGS analysis supports previous observations suggesting that PC-3 cells undergo major genetic alterations that potentially contribute to their aggressive phenotype (Seim et al., 2017). In particular, the loss of the Y chromosome in PC-3 cells has been previously associated with the ability of PC-3 cells to form tumors in athymic nude mice (Vijayakumar et al., 2005). Further functional studies are warranted to elucidate the impact of these deletions on prostate cancer progression and to identify potential therapeutic targets.

Our transcriptomic data also highlight several notable differences between parental PC-3 cells and clone #1, some of which appear to be robust across additional *LCN2*-deficient subclones. The loss of *LCN2* correlates with altered expression of genes controlling cell adhesion, inflammatory signaling, extracellular matrix remodeling, and receptor-mediated pathways. For example, downregulated genes in clone #1 include *LAMC2*, *MUC5AC*, and *MUC5B*, each of which is implicated in structural or secretory processes that can be pivotal for tumor growth and metastasis. *LAMC2* encodes a laminin subunit affecting cell-matrix interactions and invasive potential, consistent with the reduced invasiveness we previously noted in *LCN2*-deficient PC-3 cells. Mucins such as *MUC5AC* and *MUC5B* have been linked to cancer progression and cell adhesion properties in various malignancies, and their strong suppression upon loss of *LCN2* further emphasizes the interconnected roles of extracellular scaffolding and secreted proteins. Interestingly, PC-3 cells do not express kallikrein related peptidase 3 (KLK3) commonly known as prostate-specific antigen (PSA), which represents a highly sensitive but relatively nonspecific and imprecise screening biomarker for prostate cancer (David and Leslie, 2025). This finding confirms previous reports showing that PC-3 cells do not express most of the kallikrein-related peptidases (Lawrence et al., 2010; Lehto et al., 2023).

Additionally, multiple genes with known or putative roles in cancer development and metastasis were altered. For instance, *SHH* (Sonic Hedgehog) signaling is critical in many tumors, while *SLPI* (Secretory Leukocyte Protease Inhibitor) encodes a protease inhibitor associated with invasion and inflammation (Jeng et al., 2020; Nugteren and Samsom, 2021). Both genes were downregulated in clone #1. On the other hand, some genes were strongly upregulated in the absence of *LCN2*, including *CNTN1* (Contactin 1), a cell adhesion molecule involved in cell-cell communication playing important roles in cancer progression and metastasis (Gu et al., 2020), and *PROKR1* (Prokineticin Receptor 1), which can modulate angiogenesis and inflammatory processes (Meng et al., 2018; Baryla et al., 2023). Notably, *PROKR1* had sharply elevated transcripts, potentially suggesting a compensatory or alternative mechanism that might sustain tumor-promoting pathways when *LCN2* is absent.

Collectively, these altered gene expression patterns support a broad role for *LCN2* in coordinating cell adhesion, stress responses, and inflammatory pathways. This aligns with previous work demonstrating that *LCN2* deficiency in PC-3 reduces invasiveness, affects F-actin cytoskeletal integrity, and lowers proinflammatory cytokine production (Schröder et al., 2022). Several studies have investigated the relationship between prostate cancer and *LCN2* showing that the expression correlates with tumor differentiation, migration, and invasion, while the knockdown of *LCN2* suppresses the growth and invasion of prostate cancer cells (Tung et al., 2013; Ding et al., 2015). Moreover, *LCN2* expression was found to be correlated with higher Gleason score, which is a system used to assess the aggressiveness of prostate cancer (Ulusoy et al., 2021). In line, *LCN2* overexpression significantly promoted tumor growth in a xenograft model (Ding et al., 2016). Additionally, *LCN2* plays an oncogenic role in castration-resistant prostate cancer (Yan et al., 2023). On a mechanistic level, *LCN2* might act as a regulatory hub that ensures adaptation to stress and supports the expression of specific extracellular and membrane-associated proteins crucial for tumor aggressiveness. Conversely, targeting *LCN2* may open therapeutic opportunities that weaken tumor cells’ resistance to stress and disrupt their metastatic abilities.

The validity of PC-3 cells as a longstanding tool in prostate cancer research is well established. They represent a late-stage, androgen receptor-negative phenotype, harbor clinically relevant genetic aberrations (e.g., *PTEN* loss, *TP53* mutations), and exhibit robust metastatic behavior in experimental models (Fraser et al., 2012; Chappell et al., 2012; Seim et al., 2017). Their widespread employment in drug screening and *in vivo* metastasis assays has led to critical insights into the molecular underpinnings of prostate cancer progression. Our data support the continued use of PC-3 not only in the context of late-stage disease but also in more specialized applications where specific gene knockouts, such as *LCN2*, can elucidate discrete signaling cascades and test novel therapies.

*LCN2*-deficient derivatives add value to this model by clarifying how lipocalins modulate malignant properties. Clone #1 and other *LCN2* knockout subclones can be used to investigate changes in intracellular signaling, adaptational stress responses, and tumor-immune interactions. For instance, they could help assess sensitivity to chemotherapeutics or immunotherapeutic strategies that rely on heightened cell stress or inflammation. Because *LCN2* is also linked to metabolic pathways, these clones might serve to reveal how prostate tumors adapt or fail to adapt to metabolic pressures. Finally, comparing parental PC-3 cells to *LCN2*-deficient subclones provides an excellent system for preclinical evaluation of drug candidates that target lipocalin-associated pathways and for identifying potential molecular vulnerabilities created by the loss of *LCN2* function.

The complex karyotypic changes and broad transcriptomic alterations observed in LCN2-deficient PC-3 cells suggest that loss of LCN2 may modulate cellular responses to diverse therapeutic agents, including cytotoxic chemotherapies, androgen-axis-targeted drugs, and other precision therapies. This notion is supported by our recent findings that LCN2-deficient clones display markedly altered susceptibility to oncolytic viruses (Barer et al., 2023) as well as differential responses to endoplasmic reticulum stress and the unfolded protein response (Schröder et al., 2022), indicating broad effects of LCN2 loss on stress and survival pathways. Accordingly, systematic drug-response profiling of this LCN2-deficient PC-3 model, encompassing short-term viability assays and long-term clonogenic survival analyses across a panel of clinically relevant agents, represents an important future direction to elucidate the therapeutic implications of LCN2 loss in prostate cancer.

In summary, our comparative analyses of PC-3 and clone #1 highlight that both lines share key genetic hallmarks but also exhibit measurable differences in genomic stability, morphological traits, and gene expression. The numerous differentially expressed genes identified here shed light on various functional categories, many of which directly or indirectly impact tumor-related processes such as invasion, immune evasion, and growth factor signaling. These findings confirm the significant and pleiotropic role of *LCN2* in determining the aggressive features of PC-3 cells and establish *LCN2* knockouts as valuable model systems for uncovering new aspects of prostate cancer biology.

## 5 Conclusion

Our investigation of a newly generated *LCN2*-deficient PC-3 subclone, designated clone #1, highlights the complex roles of *LCN2* in prostate cancer biology. STR profiling confirms the close genetic relationship between this CRISPR/Cas9-derived subclone and its parental PC-3 lineage, while targeted disruption of *LCN2* results in noticeable changes in both gene expression and cellular phenotypes. Specifically, the absence of *LCN2* is associated with altered expression of genes that control adhesion, extracellular matrix composition, transporter activity, and inflammatory signaling. These changes in gene expression align with previous findings indicating reduced invasiveness, compromised cytoskeletal organization, and increased stress sensitivity in *LCN2*-deficient cells. The PC-3 LCN2-KO line shared about 71% of the identified rearrangements with the parental cell line PC-3, indicating a largely conserved chromosomal background inherited from the parental line. However, although the CRISPR/Cas9-edited cells retain many genetic abnormalities typical of PC-3, they do show a slightly higher number of chromosomal rearrangements, suggesting increased genomic plasticity. Overall, our results confirm that *LCN2* plays a crucial role in regulating aggressive features of prostate cancer, and PC-3-based *LCN2* knockout models are valuable tools for understanding the molecular mechanisms underlying late-stage prostate cancer. Future research utilizing these models, either alone or in conjunction with other gene manipulations, may help further elucidate the signaling pathways connecting *LCN2* to aggressiveness, ultimately aiding in the development of targeted therapies for advanced disease.

## Data Availability

The data presented in the study are deposited in the Gene Expression Omnibus (GEO) repository, accession number GSE324384.
