# Supplementary material for: Functional and genomic characterization of LCN2-deficient PC-3 cells reveals insights into prostate cancer progression
Source: Front Mol Biosci. 2026 Apr 20;13:1730948. doi: 10.3389/fmolb.2026.1730948 (PMC13136868; doi:10.3389/fmolb.2026.1730948)
Supplement: Supplementary file 1 [file Supplementaryfile1.docx]

Supplementary Material

## Supplementary Tables

**Supplementary Table 1: Primer used in this study**

| **Gene** | **Accession No.** | **Amplicon size (bp)** | **Forward primer (5'->3')** | **Forward primer position (bp)** | **Reverse primer (5'->3')** | **Reverse primer position (bp)** | **Reference/Source** |
| --- | --- | --- | --- | --- | --- | --- | --- |
| *ACHE* | NM_001302621.3 | 123 | GTTCTCCTTCGTGCCTGTGGTA | 1091-1112 | ATACGAGCCCTCATCCTTCACC | 1214-1193 | Blohberger et al., 2015 |
| *ADAM23* | NM_003812.4 | 191 | CCACTCGATTCCAAGGGTAA | 2544-2563 | ACCAGCGATGGAGCCTATTA | 2735-2716 | Ota et al., 2016 |
| *ADGRB3* | NM_001704.3 | 71 | TGCCTGTGCTGGTGTCTTAG | 667-686 | GCAGTCTCTTCCACTGTTCTTC | 737-716 | Primer BLAST |
| *AMIGO2* | NM_001370299.1 | 111 | CAAGAGTGACAGACACGGACA | 407-427 | TCCTCTTTCCTGGGTTAGTCG | 518-498 | Limbeck et al, 2018 |
| *ANO9* | NM_001347882.2 | 154 | AGGACTTCCAGGACCCTGAT | 2147-2166 | CACGTGCTCAAAGAGGATGA | 2301-2282 | Li et al., 2015 |
| *ATP2A3* | NM_005173.4 | 181 | TGCTGACCTCCGCCTCATCGAGA | 626-648 | CCACCGCTTTGCCCGATGTGA | 807-787 | Gao et al., 2021 |
| *BHLHE41* | NM_030762.3 | 72 | GCTTTACAGAATGGGGAGCG | 592-611 | AAATCCCGAGTGGAACGCAT | 663-644 | Primer BLAST |
| *CLDN1* | NM_021101.5 | 97 | TTCTTCTTGCAGGTCTGGCT | 617-636 | CTGGCATTGACTGGGGTCAT | 713-694 | Primer BLAST |
| *CNTN1* | NM_001843.4 | 169 | CAGCCCTTTCCCGGTTTACAA | 437-457 | TGCTTCTGACCATCCCGTAGT | 606-586 | Xu et. al., 2022 |
| *CXCL2* | NM_002089.4 | 85 | CTGCTCCTGCTCCTGGTG | 136-153 | AGGGTCTGCAAGCACTGG | 221-204 | Sanzari et al., 2009 |
| *CXCL3* | NM_002090.3 | 106 | CCAAACCGAAGTCATAGCCAC | 288-308 | TGCTCCCCTTGTTCAGTATCT | 394-374 | Ruan et al., 2019 |
| *CXCL5* | NM_002994.5 | 100 | CAGACCACGCAAGGAGTTCA | 253-272 | TCTTCAGGGAGGCTACCACT | 352-333 | Primer BLAST |
| *CXCL6* | NM_002993.4 | 70 | AAGGTGGAAGTGGTAGCCTC | 302-321 | TTAGAAAAGGGGCTTCCGGG | 371-352 | Primer BLAST |
| *DPP4* | NM_001935.4 | 152 | AAAGGCACCTGGGAAGTCATCG | 1429-1450 | CAGCTCACAACTGAGGCATGTC | 1581-1560 | #HP205702 |
| *EHF* | NM_012153.6 | 122 | ACCTTCCTGTTGCAGAGTCA | 672-691 | CAAGAGGATGTCGCGGATGA | 793-774 | Primer BLAST |
| *FGF12* | NM_004113.6 | 76 | CATCTTCCGGCCAACTTCCA | 138-157 | TGAGCTGGGGTTCTTTGCTC | 213-194 | Primer BLAST |
| *GABRA3* | NM_000808.4 | 118 | GCCCGTACAGTCTTTGGTGT | 1112-1131 | AGACGGCTATGAACCAGTCC | 1229-1210 | Primer BLAST |
| *GPR37* | NM_005302.5 | 79 | ACGAGCTGACCAAGAAGTGG | 1778-1797 | ACTCCCAGAGAAGCGACCTCTA | 1856-1835 | Primer BLAST |
| *HS3ST3A1* | NM_006042.3 | 108 | TTCTTCGACCGCAGCTACGACA | 1359-1380 | GCGTGACGAAGTAACTGGGCG | 1467-1447 | #HP209061 |
| *IGF2* | NM_000612.6 | 243 | AGAAGCACCAGCATCGACTT | 963-982 | AGAAGCACCAGCATCGACTT | 1205-1186 | Geßner et al., 2021 |
| *IGFBP5* | NM_000599.4 | 151 | CGAGCAAGTCAAGATCGAGAGA | 1095-1116 | TCTGCGGTCCTTCTTCACTG | 1245-1226 | Primer BLAST |
| *JPH1* | NM_001317830.2 | 130 | AATTAGGAAAGCCCCATCCG | 2244-2263 | AAGGCAGCTGTTGACTTCCA | 2374-2355 | Wan et al., 2022 |
| *LAD1* | NM_005558.4 | 105 | CACGGCCATACGGAGATCAG | 1254-1273 | TTCTCAAAGAGGTGGCGCTT | 1358-1339 | Primer BLAST |
| *LAMC2* | NM_005562.3 | 227 | GGAAAGGAAGGAGCTGGAGT | 3269 -3288 | TGTTGATCTGGGTCTTGGCT | 3495-3476 | Cave et al., 2022 |
| *LCP1* | NM_002298.5 | 183 | GCTCTTGCTGAGGTGGGCTAAT | 895-916 | TCTCCTTTTGGAGCCACCTGCT | 1023-1002 | #HP206022 |
| *MEST* | NM_002402.4 | 184 | AGATCGCCTCCGCAGGAT | 241-258 | CACACCCACAGAGTCTTGGTA | 424-404 | Primer BLAST |
| *MMRN1* | NM_007351.3 | 73 | CCTCATTGGATTGGAGGTGCT | 912-984 | CTTTGCTGTTCCTGGGCTCTT | 984-964 | Primer BLAST |
| *MUC5AC* | NM_001304359.2 | 214 | AAGTGTGCCTGCGTCTACAA | 1224-1243 | CAGGGCTTGGTCAGCACATA | 1438-1419 | Primer BLAST |
| *MUC5B* | NM_002458.3 | 304 | TGTTCACACCCTCGAGCTTC | 1611-1630 | CCGGGCGTAGTTCTCATTCT | 1915-1896 | Primer BLAST |
| *NPY1R* | NM_000909.6 | 353 | TATACCACTCTCCTCTTGGTGCTG | 897-920 | CTGGAAGTTTTTGTTCAGGAACCCA | 1250-1226 | Liu et al., 2015 |
| *P3H2* | NM_018192.4 | 169 | TTTTCCGCTCCTTGTTGGGG | 492-511 | TCGAGCTGGTTAAGCTTGATGT | 660-639 | Primer BLAST |
| *PI3* | NM_002638.4 | 87 | TCGTTCCCCAGTGAGAGGGA | 362-381 | CAGCAGGGACTTAGGACCAG | 448-429 | Primer BLAST |
| *PLEKHA7* | NM_001329630.2 | 168 | GGCGAAGCCCAAAGTTGAAG | 2743-2762 | TCTCGCTTCCTCTCGTCTGA | 2910-2891 | Primer BLAST |
| *PLXNA4* | NM_020911.2 | 460 | ATCTCCGTCTCTCAGTACAA | 1837-1856 | GTGATAGGCTTGATCACCTC | 2297-2278 | Cho et al., 2022 |
| *PROKR1* | NM_138964.4 | 73 | GCCATCGCCATTGACAGGTAT | 899-919 | CAGTGGCTGTTTGGCACTTC | 971-952 | Primer BLAST |
| *PTGFRN* | NM_020440.4 | 161 | TCCTGTCGTTGGCTCTTTGC | 326-345 | GCTGCTCCCCAAAGATGAGA | 486-467 | Primer BLAST |
| *RUNX3* | NM_004350.3 | 135 | GACTGTGATGGCAGGCAATG | 729-748 | AGGGTGAAACTCTTCCCTCG | 863-844 | Primer BLAST |
| *SERPINB2* | NM_001143818.2 | 123 | ACCCCCATGACTCCAGAGAACT | 280-301 | GAGAGCGGAAGGATGAATGGAT | 403-382 | Lee et al., 2019 |
| *SESN3* | NM_001271594.2 | 191 | GAGGATGTTGACACAACCATGCTG | 1238-1261 | CCGCCAGTAACTATCATACATGCG | 1429-1406 | Rupp et al., 2017 |
| *SHH* | NM_000193.4 | 120 | TCTCCAGAAACTCCGAGCGA | 538-557 | ACTTGTCCTTACACCTCTGAGTC | 657-635 | Primer BLAST |
| *SLPI* | NM_003064.4 | 149 | ACCCCAAACCCAACAAGGAG | 253-272 | ACGCAGGATTTCCCACACAT | 401-382 | Primer BLAST |
| *SORCS2* | NM_020777.3 | 147 | CGGGAGCCTTCATCCTCTAC | 3390-3409 | CGGGAGCCTTCATCCTCTAC | 3536-3518 | Primer BLAST |
| *ST14* | NM_021978.4 | 80 | CCTAGGATGAGCAGCTGTGG | 1201-1220 | GGGTAGTGGCCTGGGTAGTA | 1280-1261 | Primer BLAST |
| *TNS1* | NM_001387777.1 | 173 | CCATGAGCTGGTCAGGCATT | 4995-5014 | CATCTGTGGGGTCTCGGTTT | 5167-5148 | Primer BLAST |
| *UBE2QL1* | NM_001145161.3 | 231 | AACACCGAGTTCATCCTGCT | 177-196 | TTTTCTACAGATCCGTCCCTGGC | 407-385 | Primer BLAST |
| *VCAN* | NM_004385.5 | 113 | AGGTGGTCTACTTGGGGTGA | 1234-1253 | TGGTTGTAGCCTCTTTAGGTTT | 1346-1325 | Primer BLAST |

**Supplementary Table 2: Antibodies used in this study**

| **Antibody** | **Host** | **Cat. No.** | **Company** | **Dilution** | **Clonality** | **RRID^1^** |
| --- | --- | --- | --- | --- | --- | --- |
| IGF-II/IGF2 | goat | AF-292-NA | R&D Systems, Bio-Techne, Abingdon, UK | 1:500 | polyclonal | AB_354449 |
| LAMC2 (E9F7M) | rabbit | 53884 | Cell Signaling Technology, Leiden, The Netherlands | 1:500 | monoclonal | AB_3717526 |
| β-actin | mouse | A5441 | Sigma-Aldrich, Steinheim, Germany | 1:10,000 | monoclonal | AB_476744 |
| Goat anti-rabbit IgG (H+L), HRP | goat | 31460 | Invitrogen, Thermo Fisher Scientific, Schwerte, Germany | 1:5,000 | polyclonal | AB_228341 |
| Mouse anti-goat IgG (H+L), HRP | mouse | 31400 | Invitrogen | 1:5,000 | polyclonal | AB_228370 |
| Goat anti-mouse IgG (H+L), HRP | goat | 31430 | Invitrogen | 1:5,000 | polyclonal | AB_228307 |

^1^Data was collected from the Research Resource Identifier (RRID) portal, which can be accessed at https://www.rrids.org/. Abbreviations used: g, goat; HRP, horse-radish peroxidase; m, mouse; mAb, monoclonal antibody; pAb, polyclonal antibody; r, rabbit.

**Supplementary Table 3: Short tandem repeat profiling of PC-3 and PC-3 LCN2-KO cells using 15 human-specific STR loci and the Amelogenin locus**

| **Marker name** | **Cytogenetic location^1^** | **PC-3** | **PC-3 LCN2-KO** | **ATCC CRL-1435** | **Masters et al., 2001** | **van Bokhoven et al., 2003** | **Lorenzi et al., 2009** | **Fang et al., 2011** | **Yu et al.,**  **2015** | **Azari et al.,**  **2007** |
| --- | --- | --- | --- | --- | --- | --- | --- | --- | --- | --- |
| AMEL | Xp22.1–22.3 and Y | X | X | X | X | X | X | X | X | X |
| CSF1PO | 5q32 | 11 | 11 | 11 | ND | NN | 11 | 11 | 11 | 11 |
| D13S317 | 13q31.1 | 11 | 11 | 11 | ND | 11 | 11 | 11 | 11 | 11 |
| D16S539 | 16q24.1 | 11 | 11 | 11 | ND | NN | 11 | 11 | 11 | 11 |
| D18S51 | 18q21.33 | 14, 15 | 14, 15 | NN | 14, 15 | 14, 15 | 14, 15 | 14, 15 | 14, 15 | 13, 14 |
| D21S11 | 21q21.1 | 29, 31.2 | 29, 31.2 | ND | 29, 31.2 | 29, 31.2 | 29, 31.2 | 29, 31.2 | 29, 31.2 | 29, 31 |
| D3S1358 | 3p21.31 | 16 | 16 | ND | ND | 16 | 16 | 16 | 16 | 16 |
| D5S818 | 5q23.2 | 13 | 13 | 13 | ND | 13 | 13 | 13 | 13 | 13 |
| D7S820 | 7q21.11 | 8, 11 | 8 | 8, 11 | ND | 8, 11 | 8, 11 | 8, 11 | 8, 11 | 8 |
| D8S1179 | 8q24.13 | 13 | 13 | ND | 13 | 13 | 13 | 13 | 13 | 13 |
| FGA | 4q31.3 | 24 | 24 | ND | 24 | 24 | 24 | 24 | 24 | 24 |
| Penta_D | 21q22.3 | 9 | 9 | ND | ND | ND | ND | ND | 9 | ND |
| Penta_E | 15q26.2 | 10, 17 | 10, 17 | ND | ND | ND | ND | ND | 10, 17 | ND |
| TH01 | 11p15.5 | 6, 7 | 7 | 6, 7 | 6, 7 | ND | 6, 7 | 6, 7 | 6, 7 | 6, 7 |
| TPOX | 2p25.3 | 8, 9 | 8, 9 | 8, 9 | ND | ND | 8, 9 | 8, 9 | 8, 9 | 8, 9 |
| vWA | 12p13.31 | 17 | 17 | 17 | 17 | 17 | 17 | 17 | 17 | 17 |

^1^ The data depicted was retrieved from the Short Tandem Repeat DNA Database (<https://strbase.nist.gov/>). Differences in allelic variants are marked in red.

**Supplementary Table 4A: Chromosomal rearrangements found in human cell line PC-3**

| **Metaphase** | **Number of Chromosomes** | **t(8;12;1;10)** | **t(8;2)** | **t(17;15;2)** | **t(3;10;1;10;1;10;1;3)** | **t(3;17;15;1;10)** | **t(3;1;18)** | **t(4;6)** | **t(4;10)** | **t(4;12)** | **t(5;19;1)** | **t(5;19;10;11)** | **t(5;19;15)** | **t(7;18)** | **t(17;10;1;10;1;10)** | **t(11;3)** | **t(7;11)** | **t(8;12)** | **t(12;20)** | **t(16;14)** | **t(15;5;20)** | **t(15;1;10;18;10;1;15)** | **t(17;13)** | **t(3;17;15;17)** | **t(X;2)** |
| --- | --- | --- | --- | --- | --- | --- | --- | --- | --- | --- | --- | --- | --- | --- | --- | --- | --- | --- | --- | --- | --- | --- | --- | --- | --- |
| 1a | 59,XX | + | ++ | + | ++ |  |  | + | ++ | + |  | + | + |  | + |  | + |  | + | + | + |  | + |  | ++ |
| 2a | 59,XX | + | ++ | + | ++ |  |  | + | ++ | + |  | + | + | + | + |  | + |  | + | + | + |  | + |  | ++ |
| 3a | 61,XX | + | + | + | ++ | + |  | + | ++ | + |  | + | + |  |  |  | + | + | + | + | + |  | + | + | ++ |
| 4a | 61,XX | + | ++ | + | ++ | + |  | + | ++ | + |  | + | + |  |  |  | + | + | + | + | + |  | + |  | ++ |
| 5a | 60,XX | + | + | + | ++ | + |  | + | ++ | + |  | + |  |  |  |  | + | + | + | + | + |  | + | + | ++ |
| 6a | 75,XX | + | ++ | + | +++ |  |  | + | ++ | + |  | + | + | ++ | + |  | + |  |  | + | + |  | + |  | ++ |
| 7a | 62,XX |  | ++ | + | ++ | + |  | + | ++ | + |  | + | + | + | + |  | + | + | + | + | + |  | + |  | ++ |
| 8a | 58,XX | + | ++ | + | ++ |  |  | + | ++ | + |  | + | + | + |  |  | + |  | + | + | + |  | + |  | ++ |
| 9a | 57,X | + | ++ | + | ++ |  |  | + | ++ | + |  | + | + | + | + |  | + |  | + | + | + |  | + |  | + |
| 10a | 58,XX | + | ++ | + | ++ |  |  |  | ++ | + |  | + | + | + | + |  | + |  | + | + | + |  |  |  | ++ |
| 11a | 57,XX | + | ++ | + | ++ |  |  | + | ++ | + |  |  | + |  |  |  |  |  | + | + | + |  | + |  | ++ |
| 12a | 60,XX | + | + | + | ++ |  |  | + | ++ | + |  | + | + | + |  |  | + | + | + | + | + |  | + | + | ++ |
| 13a | 62,XX | + | ++ | + | ++ |  |  | + | ++ | + |  | ++ | + |  |  |  |  | + | + | + | + |  | + | + | ++ |
| 14a | 62,XX | + | + | + | ++ | + |  |  | ++ | + |  |  | + | + |  |  |  | + | + | + | + |  | + |  | ++ |
| 15a | 60,XX | + | ++ | + | ++ |  |  | + | + | + |  | + | + | + | + |  | + |  | +++ | + | + |  | + |  | ++ |
| 16a | 60,XX |  | ++ | + | ++ |  |  | + | ++ | + |  | + | + | + | + |  |  |  | + | + | + |  | + | + | ++ |
| 17a | 59,XX | + | ++ | + | ++ |  |  | + | ++ | + |  | + | + | + |  |  | + |  | + | + | + |  | + |  | ++ |
| 18a | 61,XX | + | ++ | + | ++ |  |  |  |  | + |  |  | + |  |  |  | + | + | + | + |  |  | + | + | ++ |
| 19a | 61,XX | + | + | + | ++ | + |  | + | ++ | + |  | + | + |  |  |  | + | + | + | + |  |  | + | + | ++ |
| 20a | 61,XX | + | ++ | + | ++ | + |  | + | + | + |  | + | + | + |  |  |  | + | + | + | + |  |  | + | ++ |

**Supplementary Table 4B: Chromosomal rearrangements found in human cell line PC-3**

| **Supplementary Table 4B: Chromosomal rearrangements fond in human cell line PC-3 LCN2-KOMetaphase** | **Number of Chromosomes** | **t(8;12;1;10)** | **t(8;2)** | **t(17;15;2)** | **t(3;10;1;10;1;10;1;3)** | **t(3;17;15;1;10)** | **t(3;1;18)** | **t(4;6)** | **t(4;10)** | **t(4;12)** | **t(5;19;1)** | **t(5;19;10;11)** | **t(5;19;15)** | **t(7;18)** | **t(17;10;1;10;1;10)** | **t(11;3)** | **t(7;11)** | **t(8;12)** | **t(12;20)** | **t(16;14)** | **t(15;5;20)** | **t(15;1;10;18;10;1;15)** | **t(17;13)** | **t(3;17;15;17)** | **t(X;2)** |
| --- | --- | --- | --- | --- | --- | --- | --- | --- | --- | --- | --- | --- | --- | --- | --- | --- | --- | --- | --- | --- | --- | --- | --- | --- | --- |
| 1b | 61,XX | + | ++ | + | + | + | + | + | ++ | + | + | + |  |  |  | + |  | + | + | + | + | + | + | + | ++ |
| 2b | 57,XX |  | ++ | + | + | + | + | + | ++ | + | + | + |  |  |  | + |  | + | + | + | + |  |  |  | ++ |
| 3b | 60,XX | + | ++ | + | + | + | + | + | ++ | + | + | + |  | + |  | + |  | + | + | + | + |  | + | + | ++ |
| 4b | 101,XXXX | ++ | +++ | ++ | ++ | + |  | + | +++ | ++ | + | + |  |  |  | ++ |  |  | + | ++ | ++ |  |  | ++ | ++++ |
| 5b | 58,XX | + | ++ | + | + | + |  | + | ++ | + | + | + |  |  |  | + |  | + | + | + | + |  | + | + | ++ |
| 6b | 59,XX | + | ++ | + | + | + | + | + | ++ | + | + | + |  |  |  | + |  | + | + |  | + | + | + | + | ++ |
| 7b | 60,XX | + | ++ | + | + | + |  | + | ++ | + | + | + |  |  |  | + |  | + | + | + | + | + | + | + | ++ |
| 8b | 59,XX | + | ++ | + | + | + | + | + | ++ | + | + | + |  | + |  | + |  | + | + | + | + |  | + | + | ++ |
| 9b | 59,XX | + | ++ | + | + |  | + | + | ++ |  | + | + |  |  |  | + |  |  | + |  | + | + | + | + | ++ |
| 10b | 58,XX |  | + | + | + | + | + | + | ++ | + | + |  |  |  |  | + |  | + | + |  | + | + |  | + | ++ |
| 11b | 60,XX | + | ++ | + | + | + | + | + | ++ | + | + |  |  |  |  | + |  | + | + | + | + | + | + | + | ++ |
| 12b | 60,XX | + | ++ | + | + | + | + | + | ++ | + | ++ |  |  | + |  | ++ |  | + | + | + | + | + | + |  | ++ |
| 13b | 59,XX | + | ++ | + | + | + | + | + | ++ | + | + | + |  | + |  | ++ |  | + | + | + | + | + | + | + | ++ |
| 14b | 57,XX |  | + | + |  | + | + | + | ++ | + | + | + |  |  |  |  |  | + | + | + | + | + | + | + | ++ |
| 15b | 59,XX |  | + | + | + | + |  | + | ++ | + | + | + |  | ++ |  |  |  | + | + | + | + |  | + | + | ++ |
| 16b | 59,XX |  | ++ | + | + | + | + | + | ++ | + | + |  |  | + |  | + |  | + | + |  |  | + | + | + | ++ |
| 17b | 58,XX | + | ++ | + | + | + | + | + | ++ |  | + |  |  | + |  | + |  | + | + |  | + | + | + |  | ++ |
| 18b | 62,XX | + | ++ | + | + | + | + | + | ++ | + | + |  |  |  |  | + |  | + | + | + | + |  | + | + | ++ |
| 19b | 59,XX | + | ++ | + |  | + | + | + | ++ | + |  |  |  |  |  | + |  | + | + | + | + |  | + | + | + |
| 20b | 59,XX |  | ++ | + | + | + | + | + | ++ | + |  | + |  | + |  |  |  | + | + |  | + | + | + | + | ++ |

Occurrences of rearranged chromosomes in both cell lines were detected through SKY analysis of 20 different metaphases. Specific translocations, common and unique to each cell line, were indicated using different colors. Symbols were used to represent the number of homologous chromosomes in a single metaphase: + for 1 copy, ++ for 2 copies, +++ for 3 copies, and ++++ for 4 copies. Translocations present in both cell lines were highlighted in red, those only in PC-3 LCN2-KO were in green, and those only in PC-3 were in blue. Translocations present or absent in both cell lines were shown in black. Sporadic rearrangements observed in both cell lines were excluded from the analysis. In the rearranged chromosomes, the translocated segments were named from bottom to top based on the SKY karyotype.

**Supplementary Table 5: Elevated chromosomal rearrangements in PC-3 LCN2-KO cells relative to control PC-3 cells, detected by SKY analysis**

|  | PC-3 | PC-3 LCN2-KO |
| --- | --- | --- |
| Total number of rearrangements found in 20 metaphases | 59 | 69 |

**Supplementary Table 6: Frequency of each chromosome involved in the rearrangements**

| **Chromosome** | **1** | **2** | **3** | **4** | **5** | **6** | **7** | **8** | **9** | **10** | **11** | **12** | **13** | **14** | **15** | **16** | **17** | **18** | **19** | **20** | **21** | **22** | **X** |
| --- | --- | --- | --- | --- | --- | --- | --- | --- | --- | --- | --- | --- | --- | --- | --- | --- | --- | --- | --- | --- | --- | --- | --- |
| **Different translocations found in 20 metaphases (PC-3)** | 15 | 5 | 16 | 5 | 3 | 1 | 3 | 14 | 0 | 10 | 4 | 9 | 3 | 5 | 11 | 1 | 19 | 10 | 4 | 12 | 1 | 2 | 1 |
| **Different translocations found in 20 metaphases (PC-3 LCN2-KO)** | 19 | 5 | 11 | 8 | 7 | 2 | 5 | 18 | 0 | 12 | 9 | 15 | 5 | 6 | 15 | 10 | 10 | 15 | 2 | 5 | 1 | 0 | 1 |

**Supplementary Table 7: PC-3 mRNA expression as evaluated by NGS.**

**Supplementary Table 8: PC-3 LCN2-KO mRNA expression as evaluated by NGS.**

**Supplementary Table 9: Selected genes differentially expressed between PC-3 and PC-3 LCN2-KO as assessed by NGS**

| **Gene** | **Cytogenetic**  **Location^1^** | **Transcript** | **PC-3 TPM** | **PC-3 LCN2-KO TPM** | **TPM Change**  **(Absolute)** | **TPM Change (%)** |
| --- | --- | --- | --- | --- | --- | --- |
| *AMIGO2* | 12q13.11 | ENST00000550413.2  ENST00000266581.4  ENST00000429635.1 | 21.0866  0.535437  0 | 0  0  0.94233 | 21.0866  0.535437  -0.94233 | NA  NA  0 |
| *CLDN1* | 3q28 | ENST00000295522.4  ENST00000490800.1 | 11.4671  3.72983 | 0.553512  0 | 10.913588  3.72983 | 2071.70  NA |
| *JPH1* | 8q21.11 | ENST00000342232.5  ENST00000519947.1 | 12.6603  5.8815 | 1.22895  0.924369 | 11.43135  4.957131 | 1030.17  636.27 |
| *LAD1* | 1q32.1 | ENST00000391967.7  ENST00000367313.4 | 7.18024  0.0164975 | 0  0 | 7.18024  0.0164975 | NA  NA |
| *LCP1* | 13q14.13 | ENST00000323076.7  ENST00000442275.1  ENST00000398576.6 | 22.8324  2.38499  1.14693 | 0  0  0 | 22.8324  2.38499  1.14693 | NA  NA  NA |
| *P3H2* | 3q28 | ENST00000444866.5  ENST00000319332.10  ENST00000470925.1  ENST00000427335.6  ENST00000482780.1  ENST00000490940.1 | 5.90089  5.17084  2.04039  0.86949  0.00639311  0 | 0  0  1.082104  1.29725  0  0.253356 | 5.90089  5.17084  0.958286  -0.42776  0.00639311  -0.253356 | NA  NA  212.92  67.03  NA  0 |
| *PLEKHA7* | 11p15.2-p15.1 | ENST00000530489.5  ENST00000698836.1  ENST00000531079.1  ENST00000637162.1  ENST00000355661.7  ENST00000332954.8 | 4.15057  3.74635  2.00609  1.82836  0.0204307  0 | 0  0  0  0  0  0.224766 | 4.15057  3.74635  2.00609  1.82836  0.0204307  -0.224766 | NA  NA  NA  NA  NA  0 |
| *VCAN* | 5q14.2-q14.3 | ENST00000503923.1  ENST00000343200.9  ENST00000513016.5  ENST00000265077.8  ENST00000513960.5  ENST00000512590.6 | 4.13793  2.4951  0.458259  0.348528  0.147579  0.0000000147383 | 0  0  0  0  0  0 | 4.13793  2.4951  0.458259  0.348528  0.147579  0.0000000147383 | NA  NA  NA  NA  NA  NA |
| *CNTN1* | 12q12 | ENST00000347616.5  ENST00000552248.5  ENST00000551424.5  ENST00000348761.2 | 0  0  0  0 | 3.09478  1.28741  1.52476  0.638052 | -3.09478  -1.28741  -1.52476  -0.638052 | NA  NA  NA  NA |
| *MMRN1* | 4q22.1 | ENST00000264790.7  ENST00000508372.1  ENST00000394980.5 | 0.220925  0  0 | 0  3.65749  2.35768 | 0.220925  -3.65749  -2.35768 | NA  0  0 |
| *TNS1* | 2q35 | ENST00000446688.5  ENST00000479185.1  ENST00000495556.1  ENST00000419504.6  ENST00000682258.1  ENST00000651849.1  ENST00000615025.6 | 0  0.228718  0.00673216  0  0  0  0 | 2.63946  1.79722  4.83778  0.996075  0.981323  0.0000000513731  0.617198 | 2.63946  -1.568502  -4.83104784  -0.996075  -0.981323  -0.0000000513731  -0.617198 | 0  12.77  0.14  0  0  0  0 |
| *ANO9* | 11p15.5 | ENST00000332826.7  ENST00000524802.1  ENST00000526142.5  ENST00000532094.5 | 5.07313  4.16597  2.04237  1.44143 | 0  0  0.33634  0 | 5.07313  4.16597  1.70603  1.44143 | NA  NA  607.23  NA |
| *ACHE* | 7q22.1 | ENST00000428317.7  ENST00000411582.4  ENST00000241069.11  ENST00000302913.8  ENST00000651875.1  ENST00000442452.1  ENST00000441605.2  ENST00000419336.6 | 0.624621  0.327549  0.000171848  0.0000110978  0  0  0  0 | 0  5.89025  0  0  6.53081  2.82602  0.611306  6.15951 | 0.624621  -5.562701  0.000171848  0.0000110978  6.53081  -2.82602  -0.611306  -6.15951 | NA  5.56  NA  NA  NA  0  0  0 |
| *GABRA3* | Xq28 | ENST00000497894.1  ENST00000370314.9 | 0  0 | 2.61378  23.1587 | -2.61378  -23.1587 | 0  0 |
| *ATP2A3* | 17p13.2 | ENST00000397035.7  ENST00000576957.1  ENST00000574999.1  ENST00000572694.1  ENST00000572116.1  ENST00000570845.5  ENST00000570773.5  ENST00000397041.8  ENST00000309890.11 | 0.266239  0  0  0  0  0  0  0  0 | 0  1.19816  0.417979  0.943417  0.000857693  3.97077  0.420796  1.9784  0.889863 | 0.266239  -1.19816  -0.417979  -0.943417  -0.000857693  -3.97077  -0.420796  -1.9784  -0.889863 | NA  0  0  0  0  0  0  0  0 |
| *GPR37* | 7q31.33 | ENST00000303921.3 | 9.77988 | 0.44136 | 9.33852 | 2215.85 |
| *PLXNA4* | 7q32.3 | ENST00000321063.9  ENST00000359827.7  ENST00000423507.6  ENST00000378539.5 | 5.15285  4.66692  1.16139  0.878701 | 0.0353393  0  0  0 | 5.1175107  4.66692  1.16139  0.878701 | 14581.08  NA  NA  NA |
| *PTGFRN* | 1p13.1 | ENST00000393203.3  ENST00000496699.1 | 4.98329  3.75386 | 0.0736433  0 | 4.9096467  3.75386 | 6766.79  NA |
| *ADGRB3* | 6q12-q13 | ENST00000603207.1  ENST00000370598.6 | 0  0 | 3.60846  2.8437 | -3.60846  -2.8437 |  |
| *PROKR1* | 2p13.3 | ENST00000303786.5 | 0.259417 | 7.04122 | -6.781803 | 3.68 |
| *CXCL2* | 4q13.3 | ENST00000508487.3  ENST00000510048.1  ENST00000296031.4 | 99.9223  22.8123  6.31635 | 5.40724  3.51415  2.10834 | 94.51506  19.29815  4.20801 | 1847.94  649.16  299.59 |
| *CXCL3* | 4q13.3 | ENST00000296026.4  ENST00000502974.1  ENST00000511669.1  ENST00000510390.1 | 145.039  20.315  6.74544  3.51126 | 2.05277  0  1.84156  0 | 142.98  20.315  4.90388  3.51126 | 7065.53  NA  366.29  NA |
| *CXCL5* | 4q13.3 | ENST00000296027.5 | 92.2565 | 0.400841 | 91.855659 | 23015.73 |
| *CXCL6* | 4q13.3 | ENST00000226317.10  ENST00000515050.1 | 273.072  126.014 | 8.56719  10.0216 | 264.50481  115.9924 | 3187.42  1257.42 |
| *SHH* | 7q36.3 | ENST00000297261.7  ENST00000430104.5 | 17.5634  1.64916 | 0  0 | -17.5634  -1.64916 | NA |
| *FGF12* | 3q28-q29 | ENST00000454309.7  ENST00000450716.5  ENST00000440901.4 | 0.329236  0  0 | 2.7576  3.1161  1.13886 | -2.428364  -3.1161  -1.13886 | 11.94  0  0 |
| *BHLHE41* | 12p12.1 | ENST00000394326.2  ENST00000242728.5  ENST00000541271.1 | 39.3259  25.702  13.5489 | 0  0  0 | 39.3259  25.702  13.5489 | NA  NA  NA |
| *EHF* | 11p13 | ENST00000527001.5  ENST00000257831.8  ENST00000532302.1  ENST00000529527.5  ENST00000530312.1 | 5.34819  3.44658  0.00296558  0.000000897868  0 | 0  0.10961  0  0  3.94312 | 5.34819  3.33697  0.00296558  0.000000897868  -3.94312 | NA  3144.40  NA  NA  0 |
| *RUNX3* | 1p36.11 | ENST00000338888.4  ENST00000496967.1  ENST00000308873.11 | 0.551256  0  0 | 0.00000000313582  5.50583  13.0463 | 0.551255997  -5.50583  -13.0463 | 17579325344  0  0 |
| *PI3* | 20q13.12 | ENST00000243924.4 | 948.766 | 23.2741 | 925.4919 | 4076.49 |
| *SERPINB2* | 18q21.33-q22.1 | ENST00000299502.9  ENST00000457692.5 | 18.6099  1.05036 | 0.262081  0 | 18.347819  1.05036 | 7100.82  NA |
| *SLPI* | 20q13.12 | ENST00000338380.2 | 56.0676 | 0 | 56.0676 | NA |
| *IGFBP5* | 2q35 | ENST00000233813.5  ENST00000486341.1 | 1.23003  0 | 67.3203  11.0767 | -66.09027  -11.0767 | 1.83  0 |
| *MEST* | 7q32.2 | ENST00000341441.9  ENST00000399874.6  ENST00000462132.6  ENST00000421001.5  ENST00000475188.1 | 8.43264  4.52958  2.98389  1.9614  0 | 0  0  0  0  0.670146 | 8.43264  4.52958  2.98389  1.9614  -0.670146 | NA  NA  NA  NA  0 |
| *ST14* | 11q24.3 | ENST00000278742.6 | 5.45872 | 0 | 5.45872 | NA |
| *ADAM23* | 2q33.3 | ENST00000374415.7  ENST00000444281.1  ENST00000264377.8 | 0.210511  0  0 | 5.85292  2.42705  1.37191 | -5.642409  -2.42705  -1.37191 | 3.60  0  0 |
| *DPP4* | 2q24.2 | ENST00000360534.8  ENST00000490286.5  ENST00000676768.1  ENST00000491591.5  ENST00000678583.1  ENST00000679104.1  ENST00000678740.1  ENST00000678668.1  ENST00000678566.1  ENST00000678522.1  ENST00000677212.1  ENST00000676996.1 | 5.08197  1.25256  0.873331  0.624455  0.465979  0  0  0  0  0  0  0 | 89.0614  0.0000197419  0.279944  0  4.45605  0.0000167046  1.9051  38.9221  0.983659  4.19279  0.00000657379  0.00328016 | -83.97943  1.252540258  0.593387  -0.624455  -3.990071  -0.0000167046  -1.9051  -38.9221  -0.983659  -4.19279  -0.00000657379  -0.00328016 | 5.71  6344678.07  311.97  NA  10.46  0  0  0  0  0  0  0 |
| *HS3ST3A1* | 17p12 | ENST00000284110.2  ENST00000578576.1 | 0.398958  0 | 19.8415  12.5819 | -19.442542  -12.5819 | 2.01  0 |
| *SESN3* | 11q21 | ENST00000536441.7  ENST00000416495.6  ENST00000278499.6 | 0.113409  0  0 | 2.88091  1.32502  0.459641 | -2.767501  -1.32502  -0.459641 | 3.94  0  0 |
| *UBE2QL1* | 5p15.31 | ENST00000708565.1  ENST00000399816.4 | 0.139734  0.139734 | 15.2163  15.2163 | 15.076566  15.076566 | 0.92  0.92 |
| *LCN2* | 9q34.11 | ENST00000277480.7  ENST00000494317.1  ENST00000487719.1 | 236.846  7.74634  5.34754 | 0.709064  0  0 | 236.136936  7.74634  5.34754 | 33402.63  NA  NA |
| *LAMC2* | 1q25.3 | ENST00000264144.5  ENST00000493293.5  ENST00000461729.1 | 169.903  25.7703  1.87065 | 22.8281  9.21981  1.52334 | 147.0749  16.55049  0.34731 | 744.27  279.51  122.80 |
| *IGF2* | 11p15.5 | ENST00000416167.7  ENST00000381395.5  ENST00000381406.8  ENST00000418738.2  ENST00000381389.5 | 504.83  241.221  142.672  30.0747  4.98641 | 2.57256  6.78207  0  0  0 | 502.25744  234.43893  142.672  30.0747  4.98641 | 19623.64  3556.75  NA  NA  NA |
| *MUC5AC* | 11p15.5 | ENST00000707307.1  ENST00000621226.2 | 12.2462  12.2462 | 0.0392261  0.0392261 | 12.2069739  12.2069739 | 31219.52  31219.52 |
| *MUC5B* | 11p15.5 | ENST00000529681.5  ENST00000527802.1  ENST00000531615.1  ENST00000531082.1  ENST00000526859.1 | 166.221  18.8393  5.45174  4.95741  0 | 1.16448  0  0  0  0.444714 | 165.05652  18.8393  5.45174  4.95741  -0.444714 | 14274.27  NA  NA  NA  0 |
| *TP53* | 17p13.1 | ENST00000413465.6  ENST00000269305.9  ENST00000619186.4  ENST00000504937.5  ENST00000359597.8  ENST00000604348.6  ENST00000576024.1  ENST00000571370.2  ENST00000508793.6 | 2.69204  1.39178  1.04975  1.04975  0.407641  0  0  0  0 | 0  0.0000164791  1.3895  1.3895  0.103043  0.795369  5.69934  1.77211  0.61042 | 2.69204  1.391763521  -0.33975  -0.33975  0.304598  -0.795369  -5.69934  -1.77211  -0.61042 | NA  8445728.23  75.55  75.55  395.60  0  0  0  0 |
| *AR* | Xq12 | ENST00000513847.5  ENST00000396044.8  ENST00000374690.9 | 0  0  0 | 0.156316  0.00604374  0.241511 | -0.156316  -0.00604374  -0.241511 | 0  0  0 |
| *PTEN* | 10q23.31 | ENST00000710386.1  ENST00000710392.1  ENST00000462694.1  ENST00000416679.1  ENST00000710390.1  ENST00000700024.1  ENST00000710391.1  ENST00000686459.1  ENST00000710394.1  ENST00000693560.1 | 3.7314  3.37523  3.37523  1.70385  1.08974  0.298957  0.285352  0.285352  0.131945  0 | 3.58018  2.7729  2.7729  1.42223  0.597133  0.131053  0.176785  0.176785  0.175493  0.172285 | -0.12115  0.60233  0.60233  0.28162  0.492607  0.167904  0.108567  0.108567  -0.043548  -0.172285 | 96.86  121.72  121.72  119.81  182.50  228.12  161.41  161.41  75.19  0 |
| *GAPDH* | 12p13.31 | ENST00000229239.10  ENST00000619601.1  ENST00000496049.1  ENST00000396858.5  ENST00000466525.1  ENST00000396861.5  ENST00000474249.5  ENST00000492719.5  ENST00000466588.5  ENST00000396859.5 | 3167.76  29.4932  12.8324  8.72982  3.28259  2.60006  2.60006  0  0  0 | 3078.24  22.4491  19.1846  3.7019  2.1261  17.3024  0  3.88536  5.1001  0.0000146076 | 89.52  7.0441  -6.3522  5.02792  1.15649  -14.70234  2.60006  -3.88536  -5.1001  -0.0000146076 | 102.91  131.38  66.89  235.82  154.39  15.03  NA  0  0  0 |

^1^ Data was retrieved from the Online Catalog of Human Genes and Genetic Disorders (OMIM), which is accessible at: https://omim.org/

**Supplementary Figures**


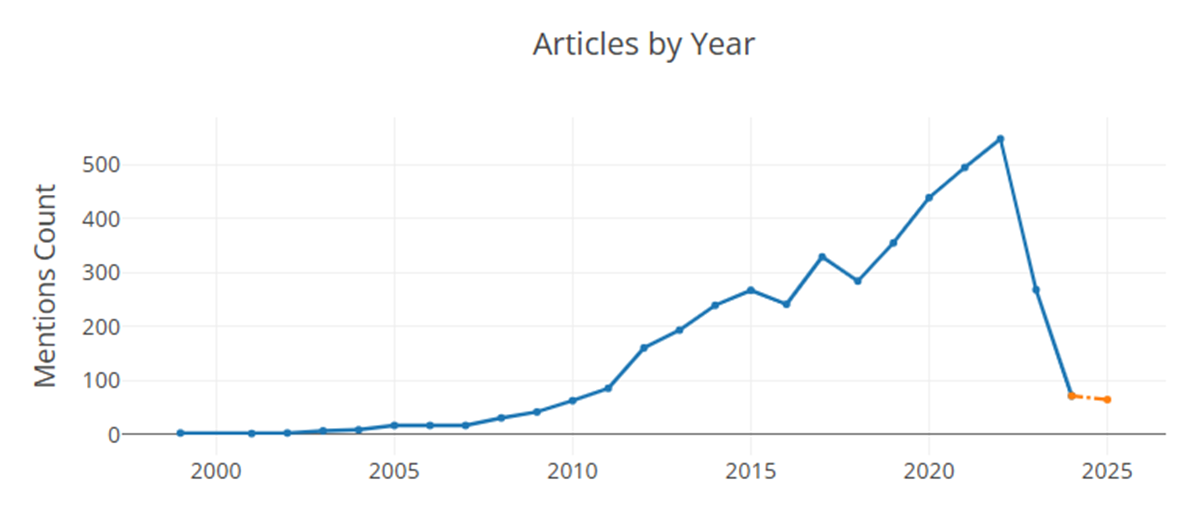


**Supplementary Figure 1: Use of PC-3 cells in biomedical research.** This image highlights the importance of PC-3 cells in biomedical research, with data sourced from SciCrunch. It is important to note that citation counts may not be entirely accurate in the most recent 1-2 years due to the time needed for SciCrunch systems to provide updates. Additionally, 65% of articles lack the proper copyright for text mining, resulting in a significant decrease in the number of identified studies. For more information on PC-3 cells, please visit: <https://scicrunch.org/resolver/CVCL_0035/mentions?q=&i=rrid:cvcl_0035-0>. The image was downloaded on October 21, 2025.


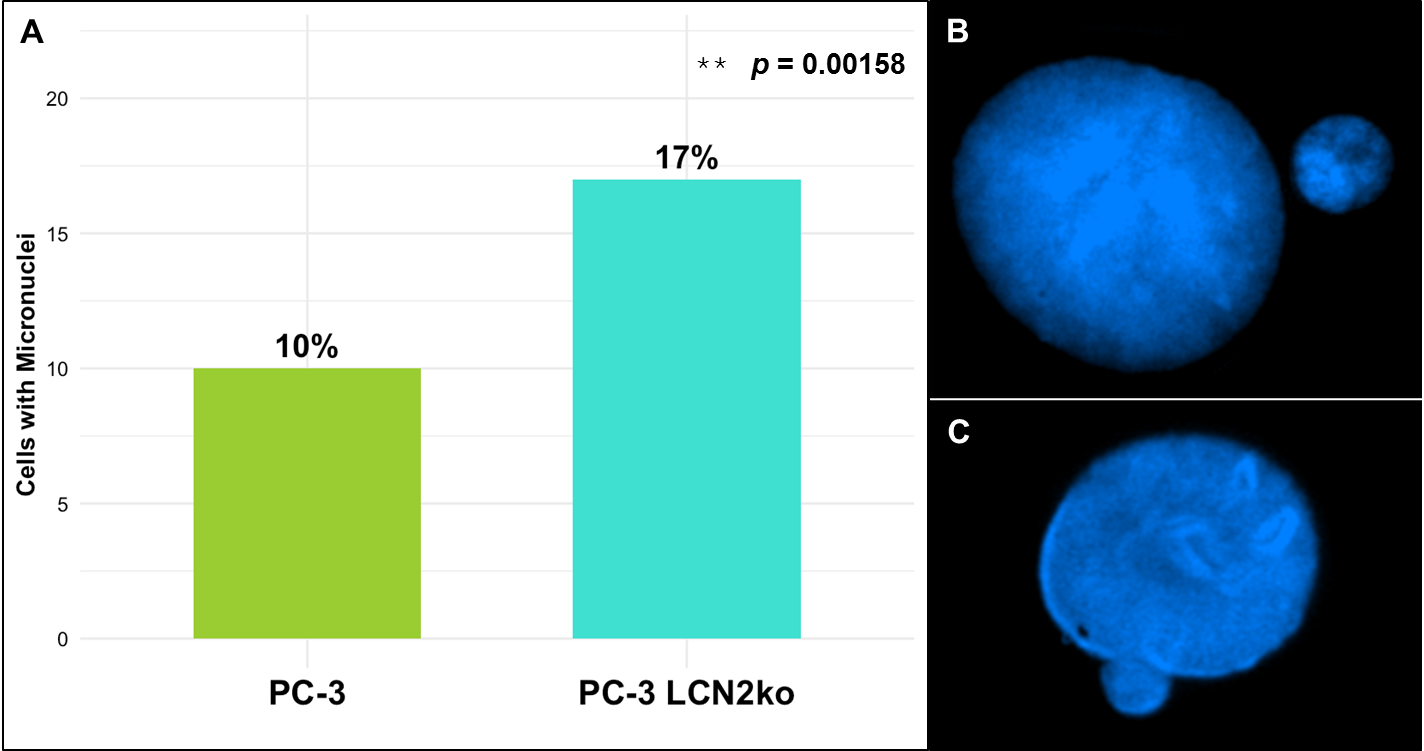


**Supplementary Figure 2**: Micronuclei formation in PC-3 LCN2-KO cells. **(A)** Histograms illustrating the spontaneous incidence of micronuclei in PC-3 and PC-3 LCN2-KO cell lines. "**" indicates a statistically significant difference in micronuclei occurrence in PC-3 LCN2-KO compared to its parental PC-3 line. **(B,C)** Representative images of micronuclei.

**References**

ATCC. Product Sheet PC-3 (ATCC® CRL-1435TM)

Azari S, Ahmadi N, Tehrani MJ, Shokri F. Profiling and authentication of human cell lines using short tandem repeat (STR) loci: Report from the National Cell Bank of Iran. Biologicals 2007;35(3):195-202. doi: 10.1016/j.biologicals.2006.10.001

Blohberger J, Kunz L, Einwang D, Berg U, Berg D, Ojeda SR, Dissen GA, Fröhlich T, Arnold GJ, Soreq H, Lara H, Mayerhofer A. Readthrough acetylcholinesterase (AChE-R) and regulated necrosis: pharmacological targets for the regulation of ovarian functions? Cell Death Dis. 2015;6(3):e1685. doi: 10.1038/cddis.2015.51

Cave DD, Buonaiuto S, Sainz B Jr, Fantuz M, Mangini M, Carrer A, Di Domenico A, Iavazzo TT, Andolfi G, Cortina C, Sevillano M, Heeschen C, Colonna V, Corona M, Cucciardi A, Di Guida M, Batlle E, De Luca A, Lonardo E. LAMC2 marks a tumor-initiating cell population with an aggressive signature in pancreatic cancer. J Exp Clin Cancer Res. 2022;41(1):315. doi: 10.1186/s13046-022-02516-w

Cho H, Park HJ, Seo YK. Induction of *PLXNA4* gene during neural differentiation in human umbilical-cord-derived mesenchymal stem cells by low-intensity sub-sonic vibration. Int J Mol Sci. 2022;23(3):1522. doi: 10.3390/ijms23031522

Fang R, Shewale JG, Nguyen VT, Cardoso H. Swerdel M, Hart RP, Futado MR. STR profiling of human cell lines: Challenges and possible solutions to the growing problem. J Forsnesic Res 2011, S2:005 doi: 10.4172/2157-7145.S2-005

Gao Y, Ma X, Wang H, Cui Y, Zhang Y, Nie M, Tong A. The Bioinformatics Analysis of Aldosterone-Producing Adenoma and Verification of Differentially Expressed Genes. Int J Endocrinol. 2021;2021:4926323. doi: 10.1155/2021/4926323

Geßner A, Koch B, Klann K, Fuhrmann DC, Farmand S, Schubert R, Münch C, Geiger H, Baer PC. Characterization of Extracellular Vesicles from Preconditioned Human Adipose-Derived Stromal/Stem Cells. Int J Mol Sci. 2021;22(6):2873. doi: 10.3390/ijms22062873

Lee NH, Park SR, Lee JW, Lim S, Lee SH, Nam S, Kim DY, Hah SY, Hong IS, Lee HY. SERPINB2 is a novel indicator of cancer stem cell tumorigenicity in multiple cancer types. Cancers (Basel) 2019;11(4):499. doi: 10.3390/cancers11040499

Li C, Cai S, Wang X, Jiang Z. Identification and characterization of ANO9 in stage II and III colorectal carcinoma. Oncotarget 2015;6(30):29324-34. doi: 10.18632/oncotarget.4979

Limbeck E, Vanselow JT, Hofmann J, Schlosser A, Mally A. Linking site-specific loss of histone acetylation to repression of gene expression by the mycotoxin ochratoxin A. Arch Toxicol. 2018;92(2):995-1014. doi: 10.1007/s00204-017-2107-6

Liu L, Xu Q, Cheng L, Ma C, Xiao L, Xu D, Gao Y, Wang J, Song H. NPY1R is a novel peripheral blood marker predictive of metastasis and prognosis in breast cancer patients. Oncol Lett. 2015;9(2):891-896. doi: 10.3892/ol.2014.2721

Lorenzi PL, Reinhold WC, Varma S, Hutchinson AA, Pommier Y, Chanock SJ, Weinstein JN. DNA fingerprinting of the NCI-60 cell line panel. Mol Cancer Ther. 2009;8(4):713-24. doi: 10.1158/1535-7163.MCT-08-0921

Masters JR, Thomson JA, Daly-Burns B, Reid YA, Dirks WG, Packer P, Toji LH, Ohno T, Tanabe H, Arlett CF, Kelland LR, Harrison M, Virmani A, Ward TH, Ayres KL, Debenham PG. Short tandem repeat profiling provides an international reference standard for human cell lines. Proc Natl Acad Sci U S A 2001;98(14):8012-7. doi: 10.1073/pnas.121616198

Ota M, Mochizuki S, Shimoda M, Abe H, Miyamae Y, Ishii K, Kimura H, Okada Y. ADAM23 is downregulated in side population and suppresses lung metastasis of lung carcinoma cells. Cancer Sci. 2016;107(4):433-43. doi: 10.1111/cas.12895

Origene Primer CD (DPP4) Human qPCR primer pair (NM_001935), Cat. No. #HP205702

Origene Primer HS3ST3A1 Human qPCR primer pair (NM_006042), Cat. No. #HP209061

Origene Primer Plastin L (LCP1) Human qPCR primer pair (NM_002298), Cat. No. #HP206022

Primer-BLAST. A tool for finding specific primers. Available at: https://www.ncbi.nlm.nih.gov/tools/primer-blast/ (last accessed October 21, 2025)

Ruan GT, Gong YZ, Liao XW, Wang S, Huang W, Wang XK, Zhu GZ, Liao C, Gao F. Diagnostic and prognostic values of C‑X‑C motif chemokine ligand 3 in patients with colon cancer. Oncol Rep. 2019;42(5):1996-2008. doi: 10.3892/or.2019.7326

Rupp M, Hagenbuchner J, Rass B, Fiegl H, Kiechl-Kohlendorfer U, Obexer P, Ausserlechner MJ. FOXO3-mediated chemo-protection in high-stage neuroblastoma depends on wild-type TP53 and SESN3. Oncogene 2017;36(44):6190-6203. doi: 10.1038/onc.2017.288

Sanzari JK, Nuth M, Kennedy AR. Induction of cytokine gene expression in human thyroid epithelial cells irradiated with HZE particles (iron ions). Radiat Res. 2009;172(4):437-43. doi: 10.1667/RR1363.1

van Bokhoven A, Varella-Garcia M, Korch C, Johannes WU, Smith EE, Miller HL, Nordeen SK, Miller GJ, Lucia MS. Molecular characterization of human prostate carcinoma cell lines. Prostate 2003;57(3):205-25. doi: 10.1002/pros.10290

Wan Y, Huang J, Song Y, Gu C, Kong J, Zuo L, Chen J. hsa-miR-340-5p inhibits epithelial-mesenchymal transition in endometriosis by targeting MAP3K2 and inactivating MAPK/ERK signaling. Open Med (Wars). 2022;17(1):566-576. doi: 10.1515/med-2022-0448

Xu S, Han L, Wei Y, Zhang B, Wang Q, Liu J, Liu M, Chen Z, Wang Z, Chen H, Zhu Q. MicroRNA-200c-targeted contactin 1 facilitates the replication of influenza A virus by accelerating the degradation of MAVS. PLoS Pathog. 2022;18(2):e1010299. doi: 10.1371/journal.ppat.1010299

Yu M, Selvaraj SK, Liang-Chu MM, Aghajani S, Busse M, Yuan J, Lee G, Peale F, Klijn C, Bourgon R, Kaminker JS, Neve RM. A resource for cell line authentication, annotation and quality control. Nature 2015;520(7547):307-11. doi: 10.1038/nature14397
